# Global Insights into Cultured Meat: Uncovering Production Processes, Potential Hazards, Regulatory Frameworks, and Key Challenges—A Scoping Review

**DOI:** 10.3390/foods14010129

**Published:** 2025-01-04

**Authors:** Renata Puppin Zandonadi, Maíra Catharina Ramos, Flavia Tavares Silva Elias, Nathalia Sernizon Guimarães

**Affiliations:** 1 Department of Nutrition, Faculty of Health Sciences, University of Brasilia (UnB), Campus Darcy Ribeiro, Asa Norte, Brasilia 70910-900, DF, Brazil; 2 Program of Evidence for Health Policy and Technologies (PEPTS), Fiocruz Brasília, Oswaldo Cruz Foundation (Fiocruz), Asa Norte, Brasilia 70904-130, DF, Brazil; maira.ramos@fiocruz.br (M.C.R.); flavia.elias@fiocruz.br (F.T.S.E.); 3 Department of Nutrition, Nursing School, Federal University of Minas Gerais, Alfredo Balena Avenue, 190, Room 314, Santa Efigênia, Belo Horizonte 30130-100, MG, Brazil; nasernizon@gmail.com

**Keywords:** cell-based meat, hazards, safety, legislation, laboratory meat

## Abstract

This scoping review aims to understand the cell-based meat production process, including the regulations, potential hazards, and critical points of this production. This review includes studies on cultured meat production processes, health hazards, and regulatory guidelines, excluding those without hazard analysis, incomplete texts, or studies published before 2013. The search was performed in eight electronic databases (MEDLINE, Web of Science, Embase, Cochrane Library, Scopus, LILACS, and Google Scholar) using MeSH terms and adaptations for each database. The search for local studies on regulations and guideline documents was complemented by a manual search on the websites of governments and regulatory agencies from different regions (e.g., FDA, FAO, EFSA, USDA, Health Canada, EC, EU, ANVISA/Brazil, MAPA/Brazil, FSANZ, and SFA). This step involved reading full texts to confirm eligibility and extract key data, including author, year, country, study design, objectives, results, cultured meat protocols, health hazards, and hazard control measures, followed by data analysis. A comprehensive search of the databases yielded 1185 studies and 46 regulatory or guidance documents. After removing duplicate studies and applying eligibility criteria to titles, abstracts and full texts, 35 studies and 45 regulatory or guidance documents were included. The cultured meat production protocols are well-established, highlighting potential hazards and critical control points. Although guidance documents and regulations are limited, they are expanding globally. The development and commercialization of cultured meat require clear, and up-to-date regulations and supervision, which are being studied and formulated by regulatory agencies worldwide. Cultured meat production presents some potential hazards (chemical, biological, and physical) that require food safety considerations: (i) genetic stability of cells/cell lines; (ii) microbiological hazards related to cell lines; (iii) exposure to substances used in the production process; (iv) toxicity and allergenicity of the product or its component for the population; (v) post-harvest microbiological contamination; (vi) chemical contamination/residue levels; and (vii) nutritional aspects/risks. Currently, no standardized testing approach exists for cultured meat. However, effective hazard and safety assessment strategies, such as HACCP combined with best practices, should be implemented throughout the production process.

## 1. Introduction

Meat is a food widely present in the eating habits of Western populations, being responsible for providing several essential nutrients, including proteins, fats, and minerals such as iron and zinc, and vitamins A and B12 [1]. Worldwide consumption of around 34 kg of meat per capita is estimated [2]. However, its production can cause harm to the local environment and sustainability since it requires the concomitant use of a series of resources, such as land, water, and energy, as well as the emission of polluting gases, such as methane. It is estimated that the production of 200 g of beef involves the use of 792 L of drinking water, 4 kg of grains for feed, the deforestation of 6.6 m^2^, and the emission of 50 kg of CO_2_ into the atmosphere [3]. Therefore, reducing its consumption is encouraged, boosting the market for meat substitutes [2]. Furthermore, there has been an increase in the demand for substitutes for foods of animal origin due to health, cultural, ethical, and environmental reasons [4,5,6].

Until recently, meat substitutes were those characterized by plant-based products (mainly using legumes and cereals), developed to present characteristics similar to those of animal-based foods, especially from a sensory point of view [7,8,9]. However, despite the industry’s advances in producing plant-based foods and the variety of products available on the market, these products still do not entirely mimic their counterparts’ nutritional, sensory, and technological characteristics, resulting in low acceptance of these products [10,11,12]. In this context, and given the influence of food on human social interactions, the habit of meat consumption and the unique characteristics that meat presents, the search for substitutes with adequate technological, sensory, and nutritional characteristics increases [1,13]. Given the above, the production of cultured meat emerges as an alternative.

Cultured meat is produced in a controlled environment through technological tissue engineering in which animal muscle stem cells are cultivated in a suitable medium for growth [6,14]. It has become an alternative to that obtained through the slaughter of animals from traditional livestock farming in future global markets in the meat supply chain [15,16]. Animal cell culture technology involves the controlled growth of animal cells, their subsequent differentiation into various cell types, and their collection and processing into food. Once produced, the collected cells can be processed or combined with other ingredients and marketed like conventionally produced beef, pork, goat, poultry, and seafood, among others [17].

Those involved in the cultured meat production process highlight potential benefits of using these products concerning their counterparts, such as less environmental damage, the absence of cruelty to animals, better nutritional quality of the product, the prospect of low cost of the products, the prospect of large-scale production to meet the growth of the population and, consequently, the demand for food [4,15,18,19,20,21]. However, there are still many doubts about the potential hazards involved in producing cultured meat and its impact on the population. The goal of producing cultured meat is to provide enough meat for the population by recreating the complex muscle structure of livestock. Cultured meat is already being sold in Singapore [22], while guidance and regulatory documents are being developed to ensure the safety of this type of food product for consumers in several countries [22,23,24,25,26,27].

The production of cultured meat is a multifaceted process that encompasses several stages, each involving its unique challenges and complexities. From cell selection and characterization, cell bank maintenance, bioreactor operation, and delivery of a safe final product, each process step is fraught with potential hazards that require careful attention and resolution [28,29]. Furthermore, given the recent growth in the number of individuals seeking alternatives to animal-based foods and the fact that this is a topic that is still largely unexplored in the scientific literature, as well as the growing interest of the food industry in developing products using this technology, it is appropriate to consider what actions, if any, may be necessary to ensure the safe production and consumption of these products. Therefore, there is a need to evaluate cultured meat production processes, the potential hazards associated with this production, and the actions to control the hazard. Therefore, to address this gap, this review aims to understand the process of cultured meat, the potential hazards (and actions to prevent or control it), and the regulations associated with this production to support actions for developing food regulation and surveillance for health protection.

## 2. Materials and Methods

### 2.1. Study Design and Search Strategy

This is a scoping review conducted following the Joanna Briggs Institute guidelines [30] and was performed according to the checklist provided by the Preferred Reporting Items for Systematic Reviews and Meta-Analyses Extension for Scoping Reviews (PRISMA-ScR) [31]. This scoping review sought to answer the following research question: “What are the processes of meat production through cell culture, regulations, potential associated hazards, and critical points in this production?”. To carry out this study, the following steps were followed: (1) identification of the research question; (2) identification of relevant studies; (3) selection of studies; (4) data mapping; and (5) analysis, reporting, and discussion of the results found. The scoping review protocol was previously registered on the Open Science Framework platform to ensure transparency, identify ongoing reviews, and avoid unnecessary duplication of research on the website https://osf.io/h3c59 (registered on 17 September 2024).

To improve the organization of our research question and the inclusion and exclusion criteria, we adopted the mnemonic PCC (P-Population, C-Context, C-Concept): P-meat; C-production, potential hazards, critical points, and regulation; C-cell culture.

The studies were searched in the following databases: MEDLINE [by PubMed], Web of Science, Embase, Cochrane Library, Scopus, LILACS [by Biblioteca Virtual em Saúde], Secretaria do Estado de Saúde de São Paulo [by Biblioteca Virtual em Saúde], Google Scholar (first 100 studies). For the field of regulations and guidance documents, the websites of governments and regulatory agencies from different regions were consulted (Food and Drug Administration–FDA/US; Food and Agriculture Organization–FAO; European Food Safety Authority–EFSA; United States Department of Agriculture–USDA; Health Canada; European Commission–EC; European Union–EU; Agência Nacional de Vigilância Sanitária–ANVISA/Brazil; Ministério da Agricultura e Pecuária-MAPA/Brazil; Food Standards Australia and New Zealand–FSANZ; Singapore Food Agency–SFA). The regulatory and guidance documents were reported and discussed in a separate topic from the studies. The keywords used included the following: “In vitro Meat”, “Cell Based Meat”, “Cultivated Meat”, “Cultured Meat”, “Cultured Meats”, “Lab Grown Meat”, “Laboratory Grown Meat”, “Artificial flesh”, “Risk”, “Hazard”, OR “production protocol”, “production process”.

The searches were restricted to the Portuguese and English languages and, considering that the first cell-cultured meat product developed for human consumption was created in 2013, searches were conducted from 2013 onwards [32,33]. The search was conducted on 13 September 2024. The reference list of the included studies was assessed to identify additional documents not captured in the search. The search strategy is described in the Supplementary File Table S1.

### 2.2. Eligibility Criteria

The inclusion criteria were as follows: (1) studies that described cultured meat production processes, indicating potential hazards; (2) studies that presented the hazards of using cultured meat for human health; and (3) legislation, guidance documents, and regulations regarding the production of cultured meat. The exclusion criteria included studies on cultured meat improvement that did not present potential hazards, risks, or forms of control, studies that did not have full text available, such as conference abstracts, letters, tables, incomplete studies, or studies that did not meet the inclusion criteria, as well as studies published before 2013.

### 2.3. Study Selection and Data Extraction

References were imported into Zotero^®^ software for management. First, duplicates were identified and removed using Zotero^®^ software. Afterward, the study selection process was conducted in two stages: (i) title and abstract review, and (ii) full-text review. The selection process, including title, abstract, and full-text reading, was carried out by R.P.Z. and N.S.G.

The data handling and summarization process followed a systematic approach to ensure clarity and consistency. During the data extraction phase, relevant information from the articles was charted based on predefined categories, including the surname of the first author (or regulatory agency), year of the study/regulation, country of origin of the paper, study design, objectives and results of the studies, cultured meat production protocols, potential health hazards of cultured meat production, and types of process control measures to avoid the hazard.

The extracted data were then organized into thematic categories to address the research question, focusing on the following: (i) cultured meat production processes; (ii) potential hazards associated with cultured meat for human health; (iii) safety assessment strategies and actions in cultured meat production; (iv) regulations and guidance documents on cultured meat; and (v) main bottlenecks in cultured meat production around the world. Each thematic category was analyzed and synthesized to identify key trends, critical control points, and gaps in the existing literature and regulatory approaches.

The charted data were further summarized in narrative format to provide a cohesive overview of the findings. These summaries supported the discussion and interpretation of the results, ensuring that essential insights into the safety, quality, and regulation of cultured meat were effectively communicated.

## 3. Results and Discussion

### 3.1. Study Selection

**Figure 1:**
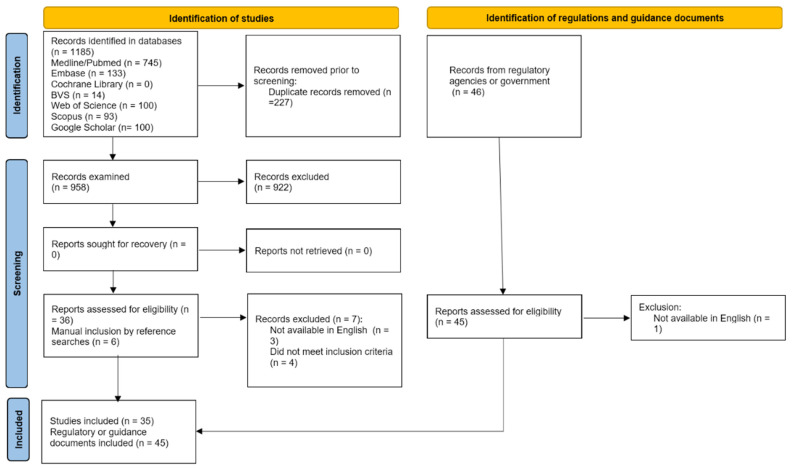
Flow diagram of the literature search and selection phases adapted from the PRISMA guidelines.

After a systematic literature search and analysis, 1185 studies were found in the databases. After the exclusion of duplicate documents (n = 227), an analysis of 958 study titles and abstracts was performed, and 36 of them were selected for full reading, considering the eligibility criteria. When reading the full texts, seven studies did not fit the scope of this review, three were excluded because they did not evaluate food safety and regulation of cultured meat, three were excluded because they presented full text in Turkish, Italian, and German, and one did not deal with cultured meat but with cured meat, resulting in a total exclusion of seven articles at this stage. Subsequently, six studies were added after thoroughly reviewing the reference lists. Therefore, 35 studies published between 2013 and 2024 were included in this review. The study identification and selection process is summarized in the flow diagram (Figure 1).

### 3.2. Characterization of Studies

The studies included in this review were carried out by researchers from 13 countries. It is worth noting that the regulations and guiding documents were entered on a different table from those of the identified studies. Most of the studies were conducted by researchers from the United States (n = 8; 23%), followed by the United Kingdom and the Netherlands (n = 5; 14%), India, Brazil, and China (n = 4; 11%), New Zealand and Italy (n = 2; 6%). And Russia, Canada, Poland, Korea, Singapore, and Belgium presented only one study each (3%). Five of the studies included (14%) were conducted by researchers from multiple countries. Of the studies included, most were reviews (n = 26; 74%), followed by experimental studies (n = 3, 8%), exploratory studies and case studies, both with two studies each (n = 2, 6%), and one report and one book (3%). More than half of the included studies were published in 2023 and 2024 (n = 9; 26% and n = 12; 34%, respectively). Table 1 presents the details of the studies, including authorship, year, country, type of study, objective, hazard associated with meat cultivated for human consumption, and measures mentioned by the study to control the hazards associated with meat cultivated for human consumption.

### 3.3. Cultured Meat Production Processes

To discuss the hazards associated with human consumption of cultured meat, it is essential to understand what it is and how it is produced. Cultured meat, also known as in vitro, lab-grown, or cell-based meat, is derived from animal stem cells grown in controlled environments and characterized by an emerging technology [6,53,54].

**Figure 2:**
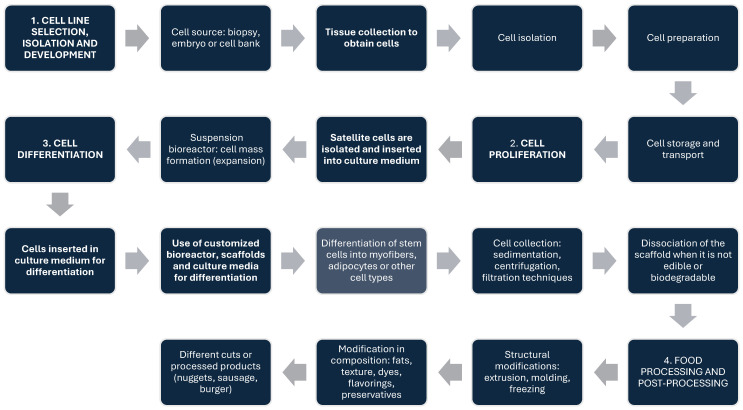
Process for obtaining cultured meat.

Cultured meat production is done by generating skeletal muscle cells (myocytes) and/or other associated cell types, including fibroblasts, adipocytes, stromal cells, vascular cells, and nerve cells, and follows general processing steps, including the following: (i) collection and development of the cell line, which involves the derivation of a source of starting cells through biopsy, isolation, storage, and appropriate treatments; (ii) proliferation, where the initial batch of stem cells is expanded to produce a maximum number of cells (increase in culture volume). In this step, cell growth occurs in a nutrient medium, including preparation and enrichment of the nutrient medium with nutrients and hormones, seeding of stem cells; (iii) cell differentiation, in which cell type-dependent biosynthesis of specific products is promoted for the desired final cell type; and (iv) processing and post-processing of food products, where structural and compositional modifications are made to produce a meat-like final product [25,50,63]. These steps are represented in Figure 2.

It is important to mention that cultured meat production involves a variety of technological routes and product forms, and Figure 2 presents one such route as it is applied in general for cultured meat. Different technologies, scopes of application, and characteristics of different product types (e.g., different meat varieties and tissue forms) exist [38,64,65,66,67,68,69,70,71,72,73,74]. All methods and technologies present strengths and weaknesses and should be carefully selected considering the meat variety, tissue type, and potential hazards. Further review could be necessary to summarize and compare different methods and technologies for different tissues and meat types, considering their strengths, weaknesses, and potential hazards. Since the basic steps of cultured meat production are similar, this review focused on the potential hazards from the usual steps shown in Figure 2.

#### 3.3.1. Collection and Development of Cell Line

The cell line development starts from a population of stem or progenitor cells obtained or generated from a tissue sample of a living or recently slaughtered animal, or from an embryo. The condition of the cells obtained may vary depending on the state of the animal from which the tissue is collected. Animal skeletal muscle consists of several cell types, such as muscle fibers, connective tissue and somatic cells, and muscle stem cells. Therefore, efficiently separating muscle stem cells from muscle tissue is important. In general, the muscle is cut into small pieces and then the muscle stem cells are purified by removing fibrous fragments, tissue, and connective tissue using proteases (e.g., trypsin or pronase) [37].

In this step, the separated muscle tissues of the animal are usually washed several times at room temperature with buffers, such as phosphate-buffered saline (PBS), to obtain primary cells from animal tissues. When collecting tissues, the age, species, and sex of the animal, as well as the location of the muscles, must be considered, as they affect the number of cells and the efficiency of the culture (e.g., the younger the animal, the greater the efficiency of the cell culture). Stem cells can also be obtained from a cell bank, provided that a stable cell line has been established and safely stored [50]. Different types of cells can be used to generate cultured meat, including adult stem cells, which are multipotent, that is, they have a more limited differentiation potential, since they create cells from the same germ layer or the same type of organ (e.g., muscle satellite cells, mesenchymal stem cells and fibrogenic/adipogenic progenitors), embryonic stem cells (they are pluripotent, that is, capable of forming any cell type), induced pluripotent stem cells, and immortalized cell lines [24,50].

Embryonic stem cells are those present in the endocytic mass of the pre-implantation embryo obtained from blastocysts. They are formed a few days after fertilization in the animal’s body or by in vitro fertilization. Obtaining the cells and the culture conditions, in addition to requiring growth factors and inhibitors of spontaneous differentiation specific to each species, represent a challenge for using this cell source [24,36].

Induced pluripotent stem cells (iPSCs) have characteristics similar to embryonic stem cells and are produced through artificial reprogramming induction, where adult somatic cells, such as fibroblasts (from the skin) or leukocytes (from the blood), undergo a reprogramming process to return to an undifferentiated state, through the use of specific transcription factors, such as Oct4, Klf4, c-Myc, and Sox2, and, subsequently, can be differentiated into cells of specific tissues, such as muscle or adipocytes [24,37]. iPSCs overcome the ethical problems and immune rejection issues caused by embryonic stem cells, but they can cause mutations in somatic cells and immunological problems and depend on studies for use in cultured meat for human consumption [37]. The process for this type of cell can present challenges related to the need for genetic manipulation, presenting the potential hazard of inserting mutations into the cell genome [24,36].

Muscle satellite cells are a type of adult stem cell that is the source of cell nuclei for producing new muscle, located between the myofibroblast and the basement membrane [28]. They are the most commonly used cells for cultured meat production because satellite cells easily differentiate into mature myotubes and myofibrils due to their regenerative capacity. The efficiency of cell culture of muscle satellite cells is relatively low [24,37]. Despite this, muscle satellite cells are still widely used because they are abundant, acquired directly from the animal’s muscles, require less effort for differentiation, and are considered the most advantageous for receiving permission for use as food [24,37]. In addition, activated muscle satellite cells play a crucial role in repairing damaged muscle tissue replacing dead or damaged cells [28].

In addition to muscle satellite cells, other types of adult stem cells are used. Mesenchymal stem cells promote differentiation into adipocytes, chondrocytes, and fibroblasts. In the case of cell-based fat creation, mesenchymal stem cells isolated from fat or bone marrow can be used, as these multipotent stem cells can develop into adipocytes [25]. Fibrogenic/adipogenic progenitor cells can differentiate into fibroblasts and adipocytes. Together, they can compose the main cell types present in meat. These cells are being tested for cultured meat production and still require optimization to increase their differentiation and proliferation capacity, reducing the need for animal biopsies to obtain them [24]. Adipose-derived stem cells (ADSCs) can also be triggered to develop into various cell types, such as bone, muscle, and fat cells [25].

For large-scale meat cultivation, it is necessary to increase the capacity for cell division, which is limited under natural conditions when the cell enters senescence. Therefore, producing immortalized cell lines is essential for proliferation and yield on an industrial scale. This occurs through epigenetic modifications (e.g., inhibition of the p38-MAPK cell signaling pathway) and expression of telomerase enzymes. The technology for producing immortalized cell lines is being improved to improve the capacity for cell passage number (cell division from one generation of cells to another), the subculture limit that is reduced over the passages, the proliferation of unidentified cells, as well as the capacity for differentiation into cell types other than myocytes (e.g., adipocytes) [24,26].

The two primary stem cells considered most suitable for meat cultivation are embryonic stem cells or satellite cells due to their ease of differentiation and close analogy to the natural cell type [6,54]. Given the need for various cell types to produce cultured meat, there is a practical advantage in using pluripotent embryonic stem cells over their multipotent counterparts derived from adult animals (e.g., satellite cells). However, as mentioned, ethical issues may arise from using embryonic stem cells, and therefore, there is still a need to evaluate stem cell sources from ethical, economic, and production perspectives [54].

For consistent production of cultured meat products, it is also vital to use stable cell lines that maintain genetic and physiological characteristics and exhibit uniform and consistent production performance over time. This requires storing cells after isolation from animals (primary cells) or from specific stages of the production process. To this end, cells are stored in master cell banks after adding cryopreservation fluid. Individual vials from the master cell bank can then be used to generate functional cell banks to initiate cultures for cultured meat production. Before cryopreservation, cell lines must be screened for the presence of microbial contaminants and can be verified for cell line species identity to ensure that cell cultures are not contaminated during the seed phase of cultured meat production [25].

#### 3.3.2. Cell Proliferation and Differentiation (Phases 2 and 3)

Cell lines must be adapted or engineered (Phase 1) to achieve desirable characteristics, such as suspension growth, enhanced proliferative rates, and immortalization. Through careful monitoring of cell growth and characteristics, a stable and consistent cell line is developed and stored in a master cell bank, with working cell banks used for subsequent processing. Subsequently, in Phase 2, large-scale cell proliferation is performed to achieve exponential cell growth by optimizing cell proliferation in progressively larger bioreactors, where the product from one bioreactor serves as the “seed” for a larger one. The process culminates with large-scale cell expansion, typically in suspension bioreactor systems [25,63]. The harvested cell mass forms the basis for differentiation and maturation, where the biosynthesis of specific products is promoted as cells are directed toward specialized cell types (Phase 3) [63].

Thus, to initiate the culture for cell proliferation, the obtained cells are placed in an ex vivo environment that is used to maintain their growth. This environment comprises three main components: (i) a sterile container, such as a plastic culture flask or bioreactor; (ii) a nutrient medium containing nutritional components to support cell growth by providing the nutrition necessary for survival and the growth factors necessary to control growth; and (iii) a scaffold which is a biocompatible material used to provide 3D mechanical support to the cells [37]. Scaffolds are porous 3D structures that function as a template for tissue formation and usually mimic the extracellular matrix for cells to attach, proliferate, or differentiate, providing physical and bio-chemical directions for the cells to adhere, proliferate, and differentiate into the different cell types. Scaffolds provide spatial heterogeneity in the cultured meat, resembling the natural meat structure and texture [75].

In the proliferation stage, stem cells grow and divide, stimulated to remain as stem cells, to increase cell number, but without differentiation (later, in differentiation, stem cells are triggered to differentiate into the desired cell type). The degree of cell proliferation depends on the species and type of cell, and cell growth and division depend on appropriate regulatory activities based on the cell cycle. The efficiency of cell cultures is usually assessed based on the degree of cell proliferation [37].

Cell types, such as skeletal muscle cells, fibroblasts, satellite cells, and iPSCs, are generally favored, either alone or in combination with adipogenic stem cells, and each has its benefits and requirements for proliferation factors, such as oxygenation, pH, and temperature. Most mammalian cells proliferate in a temperature range of 36.5 to 37.5 °C; fish cells proliferate between 15 and 30 °C and tolerate a wider pH range and lower oxygen levels than mammalian cells. Culture media is also considered a critical factor for cell growth [25].

There are different ways to control the culture and growth phases. Stem cells must be cultured in a medium suitable for the proliferation stage. Currently, animal serum-based media, particularly those containing fetal bovine serum (FBS), are the most widely used [37,54]. FBS is a serum separated from bovine blood extracted from the uterus of the cow (mother) that contains nutrients, growth factors, and hormones essential for cell culture and, despite its variable composition, is widely used due to its low rate of immunological rejection. This serum provides growing cells with essential nutrients, hormonal and differentiation factors for cell proliferation (e.g., growth hormone and insulin), transport proteins (e.g., transferrin and transcortin), and growth factors (e.g., epidermal, endothelial, fibroblast and insulin-like growth factors). However, for food safety and ethical reasons (it depends on the animal for extraction and is usually taken by puncture from fetuses up to 3 months old), it has been recommended to develop synthetic media, without animal serum, with chemically defined components, which can reduce the variability between serum batches and the standardization of the standardized culture media with commercial-scale production [24,36]. Although there are many formulations of serum-free media, growth requirements vary with cell type and, therefore, there is no universal serum-free medium for cultured meat production [28,54,76].

It is important to note that a large amount of growth factors and serum are used to maintain undifferentiated satellite cells in proliferation. A change from the culture medium used for the proliferation phase (Phase 2) to a culture medium used for cell differentiation, where there is a reduction in the amount of growth factor and serum, is necessary to trigger differentiation (Phase 3) [37]. Cell differentiation can be stimulated by changing to a culture medium with an altered composition of signaling molecules, environmental conditions, or by changing scaffolds [25]. As an alternative to modifying the culture medium to switch from the proliferation phase to the differentiation phase, there is the possibility of using a genetically modified cell line that contains a genetically inducible switch. In this way, when a chemical is added, the cells remain proliferating, while the addition of another chemical and the removal of the first will induce differentiation. However, this alternative approach must be extensively studied and evaluated to avoid potential chemical and tumorigenic hazards that are not yet fully established [28,37,54]. Future studies are needed to establish long-term effects related to tumorigenicity.

A culture medium that is safe and suitable for consumption as food is essential for producing cultured meat. The basic medium consists of glucose, glutamine, and other amino acids, vitamins, inorganic salts, signaling molecules, such as growth factors, and buffers. It also includes additional components necessary to induce the desired cellular condition, such as proliferation, attachment, and differentiation, as well as to facilitate cell survival. Table 2 lists the basic components of culture media and their respective functions.

In addition to the culture medium, an essential component in this phase is the scaffold, since most types of cells associated with muscle are adherent by nature and would die naturally without it [6,37,77]. Different types of scaffolds are used in cell culture to produce cultured meat. The simplest are microcarriers, typically used for large-scale cell proliferation. For the tissue maturation phase, scaffolds are generally categorized as porous, hydrogel, or fiber scaffolds [78,79]. Both porous and fiber scaffolds have voids through which the culture medium can circulate. However, porous scaffolds have a sponge-like structure, and fiber scaffolds have long, thin fibers (usually produced by electrospinning). Microcarriers can also belong to one of the other three categories of scaffolds, most commonly hydrogels, but due to their exclusive role as suspension culture scaffolds, they are categorized differently [78].

Scaffolds for cultured meat production not only affect the structure but can affect the texture and flavor of the cultured meat. Therefore, it is recommended that the scaffold and its byproducts be edible or biodegradable. Scaffolds can be made from animal or non-animal components, natural or synthetic, such as collagen, cross-linked pectin, agar and alginate or polylactic acid, polyacrylamide, and polyglycolic acid [37]. There are other proposals, such as using a mesh scaffold and perfusion media through the scaffold or using microcarriers, but these must be designed in conjunction with bioreactors, suitable for scalable production and to accommodate the proliferation, differentiation, and maturation of stem cells.

Comparing the scaffold method and the self-assembly method using microcarriers, scaffolds have superior muscle differentiation performance compared to microcarriers because they mimic the extracellular matrix of cells in the body. However, there is a disadvantage in that they cannot be used in a floating incubator. Therefore, microcarriers were developed to solve this problem [37]. Microcarriers are effective in scaling cell proliferation because they increase the surface area available for cells to proliferate by attaching cells to microspheres of approximately 100–300 μm in diameter. Microporous microcarriers prepared with various materials, such as dextran, gelatin, collagen, and cellulose, are applied to attached and floating cells. These microcarriers are mixed with the culture medium and cells in a 3D bioreactor, forming a microcarrier-cell complex. However, there is a challenge associated with the microcarrier culture method, as cell activity and proliferation are inhibited due to the shear stress generated in the continuous mixing process of the cell-microcarrier complex. In addition, when applying microcarriers to cultured meat, it is important to standardize the technology to form muscle tissue by differentiating mesenchymal stem cells. It is also necessary that the microcarriers be manufactured with materials that can be consumed without separating them from the formed muscles [37].

Scaffolds can be fabricated into a form that has flexible properties or can be mechanically pulled and unrolled to promote third-stage cell differentiation. When using the scaffold method, embryonic or adult cells are attached to the scaffold or microcarrier and cultured again by injecting the medium from a static culture device or a rotary culture machine. Cells cultured in this way can be used as cultured meat when myotubes are formed [37,78]. The most commonly used materials in the production of scaffolds are collagen, extracellular matrix proteins, laminin, cellulose, chitosan, polyethylene, polystyrene and epoxy, poly(glycolic acid), poly(lactic-glycolic acid), polylactic acid and poly(N-isopropyl acrylamide), gelatin, fibrin, Matrigel, and elastin, such as hyaluronic acid, agar, dextran, alginate, Cytodex (commercial microcarriers), RGD binding groups (arginylglycylaspartic acid—peptide responsible for cell adhesion), protein kinase inhibitors associated with RHO (guanine nucleotide binding proteins), and fibronectin [50].

Depending on the scaffold used, the cells may need to be removed from the scaffold, or the scaffold may be used together with the cell biomass to produce a meat product [6,37,77]. For example, a collagen/gelatin scaffold may be incorporated into the final product, whereas a polystyrene scaffold would not be suitable for consumption and would require removal. Scaffold removal can occur in several ways, such as mechanically removing the cells, enzymatically separating the cells from the scaffold, or using a scaffold material with engineered properties, such as thermal elevation or pH elevation, where a change in temperature or pH causes a reversible change in the scaffold structure that allows the cells to detach [37,79].

Different cultured meat products are likely to contain varying percentages of scaffold materials as part of the final product weight. There are cases where the scaffold may biodegrade to undetectable levels in the final product. However, in the case of scaffolds that remain integrated into the final product, food safety regulations are required as an ingredient or additive, depending on the level present and local regulatory definitions [78]. Therefore, scaffold and biomaterial suppliers must manufacture scaffolds following general HACCP principles and under good food practice conditions to avoid unintended food safety risks, such as a cross-contamination and the presence of allergens, among others. Depending on the method used to construct the scaffold, it may be contaminated by chemical solvents that are not food-safe or may include other non-food-safe components, such as some photoinitiators and chemical crosslinking agents used for scaffold polymerization [24,78].

Further research into scaffold manufacturing may be needed to avoid potentially hazardous solvents and develop food-safe polymerization agents. If a scaffold is intended to degrade or transform throughout the manufacturing process, potential degradation byproducts may require further safety assessments. Physicochemical transformations, such as oxidation, degradation, or enzymatic processing of synthetic polymers, may also affect product quality and give rise to new safety risks. Therefore, manufacturers of cultured meat should consider the safety implications of all scaffold inputs and material processing agents before their use to mitigate food safety risks [78].

The scaffolds used depend on the type of bioreactor. Bioreactors for the production of cultured meat are essential in tissue engineering, promoting ideal conditions, such as low shear stress and constant perfusion, for the production of cultured meat. Different types of bioreactors are required depending on the type of cultured meat to be produced. Bioreactors are commonly made from plastic and/or metal, and the stirred tank bioreactor is the most widely used for large-scale and economic growth of cells for food production [25,50,79]. In addition to the stirred tank bioreactor, there are other different formats or types, such as rotary format and static culture type, requiring specific scaffolds (selected based on the type of bioreactor, the format of the cultured meat, and the predominant cell culture) [37]. The scaffold for use in the production of cultured meat should have the following characteristics:

- Cell adhesion: Ideally, nearly 100% of cells should be able to attach to the scaffold.
- Permeability: To facilitate residue recovery and nutrient supply, the micropores within the scaffold must not be blocked until the last stage of culture.
- Do not inhibit muscle differentiation: Scaffolds should not affect muscle differentiation in cells.
- Bioreactor Compatibility: The scaffold must be compatible with the bioreactor as it must be placed in the bioreactor with the cells and remain in the culture medium for 3–4 weeks for cultured meat to be made.
- Edible (preferably): Scaffolds must be made of edible materials so that the product obtained by the growth of cells attached to the scaffolds can be consumed without separating them from the scaffold. If they are not edible or degradable, there is a need to separate the scaffold from the product generated.

More complex tissue structures, such as the whole steak, present greater biological complexity that requires the creation of a complex, intricate vascular network. There are projects for producing more structured tissues, such as using 3D bioprinting technologies, where cells are placed in a hydrogel and molded into shapes [50,80]. Three-dimensional (3D) printing is a technology that uses a predetermined computer-aided design template in which material is continuously extruded and adhered to previously printed layers of material, resulting in layer-by-layer printing, i.e., bio-inks mixed with living cells are printed in 3D to produce natural tissue-like three-dimensional structures [79]. This technology offers the ability to customize scaffolds according to the required pore structure and adjust their spatial structure [80]. In laboratory-based meat production, 3D bioprinting technology involves the layer-by-layer spatial arrangement and assembly of living cells alongside biological products and/or biomaterials in a prescribed organization, forming a 3D living cellular construct. This process represents a considerable challenge, as living cells must be delivered into each bioprinted layer without significantly altering their character and viability [80].

The three main categories of 3D bioprinters are extrusion, laser, and inkjet. Inkjet bioprinters have the advantage of being more affordable, more accurate, and operating with little cell disruption. However, as the scaffold size increases, the quality of the 3D stacking is compromised by the low viscosity at which inkjet bioprinters discharge the bioink, leading to a structurally less robust scaffold. Laser-based bioprinters print microscopic droplets using a laser beam and a proprietary lens, offering higher resolution than other bioprinting modalities. Laser bioprinters do not have nozzles, allowing for the printing of materials with higher viscosity, but the laser used can unintentionally damage the cultured cells. Extrusion bioprinters are manually or pneumatically propelled, producing scaffolds with robust mechanical qualities and allowing the use of bio-ink with very high viscosity. However, extrusion bioprinters produce mechanical stress at the nozzle tip, which restricts the types of biomaterials used in the bioink and results in lower final scaffold resolution than other bioprinters. Although 3D bioprinting holds promise for improving in vitro meat culture methods, technological advances and adjustments are needed to successfully employ it in large-scale protein manufacturing [79]. Table 3 presents the characteristics of the different strategies for tissue structuring.

During the differentiation and proliferation stages, it is necessary to simulate what occurs naturally under homeostatic conditions and to control cell growth, ensuring the appropriate stages of myogenesis. In all bioreactor configurations, it is important to monitor the process and control parameters, such as moisture level, dissolved O_2_ and CO_2_, temperature, waste removal, pressure, viscosity, hydrodynamic pressure, cell density, viable cells, glucose levels, flow rates, sparging rates, bubble size, waste removal, pH, and mixing speed [25,50]. There are some ways to manage these factors, such as using perfusion pumps that continuously remove waste or using sensors that monitor the physicochemical parameters of the culture and automatically rectify the necessary changes, such as adding more buffer, adding more O_2_, or increasing flow rates [50,63].

#### 3.3.3. Processing and Post-Processing of Food Products (Phase 4)

Once a sufficient level of culture volume has been obtained and the cells have differentiated and matured, they are harvested in a manner that maintains tissue integrity and processed to produce cultured meat products (Phase 4) [25,50]. It is expected that cell death will occur during cell harvesting, as occurs in conventional meat production after slaughter, and that proteolytic cell death events will also happen similarly. However, it is recommended that these events be studied, as they may occur with different intensities between the two types of meat [24]. Cells can be harvested using sedimentation, centrifugation, or filtration techniques. Cells grown on non-edible and non-biodegradable scaffolds or microcarriers must be dissociated from the scaffold before processing. Dissociation can be done by enzymatic, chemical, thermal, or mechanical methods and, depending on the production system used, only part of the cells may be harvested [25].

When temporary microcarriers or scaffolds are used in the cell proliferation phase, they must be removed for processing. To this end, they must present characteristics such as easy detachment and separation of cells. The dissociation of cells from the microcarriers or scaffolds must occur in a way that maintains cell viability, proliferation, and differentiation capacity. Dissociation by chemical means can be enzymatic and non-enzymatic. The enzymatic method is based on proteases in combination with Ca^2+^ chelating agents to reduce cell binding. Non-enzymatic dissociation uses components, such as dextran sulfate, N-acetyl-L-cysteine, and dithiothreitol, which mimic enzymatic activity by cleaving or degrading the microcarrier coating. Mechanical methods to separate cells from microcarriers include pipetting, high agitation, and vibration. They can be combined with enzymes and chelators such as trypsin-EDTA. The thermal method uses thermally responsive materials to detach cells from microcarriers or scaffolds, generating a phase transition or morphological modification in response to a temperature variation, leading to cell detachment. Mechanical and thermal methods have advantages over chemical methods, as they do not require dissociation agents and do not have washing steps before and after dissociation, reducing processing time and extensive culture manipulation [25].

After the cells have been dissociated, they must be separated by filtration, centrifugation, inertia, and magnetism. The most common methods use dead-end filtration systems, tangential flow filtration (alternative), or continuous centrifugal separators. Magnetism can be used when the core of the microcarriers or scaffolds has magnetic particles (made of iron, nickel, cobalt, or their alloys) embedded in them, and a magnetic field separates the microcarriers from the cells [25].

In the case of using degradable and non-edible degradable microcarriers, they serve as a temporary substrate for cell proliferation, and they are degraded at the end of the process instead of dissociation to obtain cells. Several natural or synthetic degradable materials have been used for this purpose, such as polystyrene, cellulose, collagen, gelatin, alginate, chitosan, poly(L-lactic-co-glycolic acid) (PLGA), polylactide (PLA), and poly(caprolactone) (PCL). These polymers can be degraded by thermal, chemical, mechanical, photodegradation, and biological degradation. Degradation needs to be controlled and rapid. It also must avoid damage or interaction of cells with degradation products. In addition, premature degradation of microcarriers should be avoided during cell proliferation. One commercially developed microcarrier designed to be fully and rapidly biodegradable for cell collection is made of cross-linked polygalacturonic acid (PGA) and is readily dissolved within 10–20 min using an EDTA solution in combination with pectinase for polymer digestion. Other polymers, including dextran, cellulose, collagen, pectin, or gelatin, can be enzymatically digested similarly [25].

Photodegradation and thermal degradation are considered the least suitable for cell culture, as the high temperatures required to thermally degrade polymers or ultraviolet radiation to induce photodegradation can cause denaturation and damage to proteins and DNA in cells. Mechanical forces, such as agitation or fluidization, can also be combined with chemical degradation (enzymatic or non-enzymatic) to facilitate the degradation process and reduce the enzyme concentration. Degradable microcarriers or scaffolds simplify the process by eliminating the need for a separation step and allowing for greater cell recovery. The cell suspension from this process can be washed and used directly for further processing [25].

When edible microcarriers or scaffolds are used, they can be incorporated into the final product. Unlike the previous processes, edible microcarriers or scaffolds must comply with the regulations for use as an ingredient or food additive in cultured meat production. The main edible polymers used in these products include the following: (i) polysaccharides, such as starch, alginate, carrageenan, chitosan, cellulose, carboxymethyl cellulose, and pectin; (ii) polypeptides, such as collagen, gelatin, and gluten; (iii) paraffin and shellac and their composites/synthetics, such as cross-linked polygalacturonic acid (PGA) and polyethylene glycol (PEG). These products have been widely used as stabilizers, thickeners, coatings, and emulsifiers in the food industry. In this case, the dissociation step can be omitted completely, and the edible polymer used as a cell substrate during cell proliferation can be designed to improve or introduce desired properties, such as texture, flavor, or color [25].

Subsequently, the obtained product can be used to produce ground products that are simpler because they have fewer structural requirements (e.g., hamburgers, nuggets, sausages, and ground meat) or more complex tissue structures (e.g., steak), which present greater structural and biological complexity [63]. Different types of cells (e.g., muscle and fat cells) can be combined to replicate the structure and texture of conventional meat or meat cells/tissues combined with plant components to produce blended products. Shear cell technology, extrusion, or 3D printing are used to obtain structure and texture in cell-based food products depending on the type of final product desired. In addition, biopolymers, such as alginate and polysaccharides, including carrageenan, pectin, gellan, and xanthan, can confer structure to cultured meat. Alginate stands out because its gelatinization can occur at low temperatures by adding or releasing calcium ions [25]. In addition, at this stage, additional formulation and processing steps are performed, such as the use of transglutaminase or binding proteins to improve the consistency and texture of the cultured meat, and the addition of nutrients, flavorings, hemoglobin, or other additives to the cell mixture to provide sensory or nutritional characteristics to the final product. Finally, the product will be packaged for commercialization [29,50].

### 3.4. Potential Hazards Associated with Cultured Meat for Human Health

At each stage of cultured meat production, different chemicals, biologicals, media formulations, additives, and supplements are used to ensure a successful culture [50,63], representing potential hazards of various natures [81]. In general, it is found that some of the control points to produce cultured meat are similar to the control points in the production of food products from traditional meat (raw materials, reception of raw materials, materials, production, packaging, and storage of the final product), but with some specificities [81].

Quality control and hazard assessment are fundamental elements in the cultured meat manufacturing process, which is indispensable in ensuring safety, reproducibility, and mitigating variability arising from heterogeneity between cell donors and all components involved in the entire cultured meat production process [28].

Given the many processes and inputs used, ensuring that what remains in the final product for human consumption does not have undesirable consequences for human health is necessary. Therefore, each stage of the cultured meat production process must be monitored to track potential sources of hazard and contamination [24]. Therefore, in the production of cultured meat, it is imperative to properly identify hazards and implement a system based on the principles of hazard analysis and critical control points (HACCP) to perform the analysis and control of biological, chemical, and physical risks at all stages, from the production of the raw material to the final product [38,49,81,82]. The studies identified in this review point to different hazards in each stage of cultured meat production (Table 4). In the first phase (obtaining, developing, transporting, and storing the cell line), 16 potential hazards were described in the studies; in the second phase (proliferation), 13 potential hazards were described; in the third phase (differentiation), 11 hazards were identified; and, in the fourth phase (processing and post-processing of the final product), 18 potential hazards were identified. For each hazard, the studies proposed several control actions (Table 4), detailed by phase of the cultured meat production process.

The potential hazards associated with the consumption of cultured meat are due to physical, chemical, and biological factors, in addition to the potential occurrence of allergic reactions and inadequacies from the point of view of the nutritional composition of the product. Each of the types of hazard identified is not specific to a particular stage of cultured meat production and can negatively affect several critical points of production and require different control measures (Table 1 and Table 4).

According to the *Codex alimentarius*, a control measure is any action that can prevent, eliminate, or reduce a hazard at an acceptable level [83]. Thus, the potential hazards associated with cultured meat production and the process and management interventions that can reduce the likelihood of their occurrence [38]. The control measures found in this review, as well as other measures to be identified in the future, need to be considered as part of the risk analysis process and in the development of a food safety management plan in cultured meat production aligned with the HACCP principles defined by the *Codex alimentarius* [83]. However, it is essential to emphasize that some potential hazards, such as metabolites formed during the production process of cultured meat, are not easily predictable but need to be identified and evaluated from a food safety perspective. Given some uncertainties in the production process of cultured meat, applying HACCP becomes essential to develop a safety management plan for this food for human consumption. Therefore, there is a need for guidance through the Regulations on the safety of these products, according to which the manufacturer of these foods must develop, implement, and comply with procedures based on HACCP [49,50,83].

#### 3.4.1. Hazards Related to Contamination During Collection, Storage, and Transportation of the Cell Line

The selection and characterization of cells for culture must prioritize safety, requiring meticulous donor selection, accurate biopsy techniques, and appropriate transportation procedures [28]. One of the potential hazards identified in the first step is the animal’s health status from which the cells will be collected. To mitigate this hazard, animals eligible for cell extraction by biopsy must be in a healthy condition and certified by a veterinarian, like the existing procedure for animals slaughtered for traditional meat production. There must be a pre-biopsy inspection by a veterinarian and documentation of any medications administered to the animal, such as antibiotics (including dates of administration and the established withdrawal period for each medication), and identification of any diseases present in the animal that could potentially affect the safety of the resulting cultured meat product [24,28].

The use of veterinary drugs (including antibiotics) present in the tissues used as cell sources may pose a potential chemical hazard in cultured meat production. To pose a hazard, the drug would need to persist throughout the cell line development, production, and food processing stages without detection, reaching the final product at a concentration that exceeds a maximum safe level [25]. This hazard can be controlled by accessing the health records of the source animals. Process controls are also in place for the withdrawal of veterinary drugs. In addition, tests should be used to quantify the levels of veterinary drugs in the cell line and in the final product. It should be noted that this hazard is not exclusive to cultured meats, as it can also be present in meat from conventional livestock and aquaculture. However, in cultured meats, veterinary drugs are considered an unintentional chemical contaminant introduced unintentionally through the cell source in the production process, whereas in animal husbandry, they are considered a residue [25].

Although contamination by bacteria of toxicological interest, such as *Salmonella*, *Listeria*, and *E. coli* is unlikely to occur in cultured meat (unlike conventional meat), because they are enteric bacteria in which contamination occurs through fecal material, care must be taken with asepsis during collection of the material [24]. To avoid exposure to contaminants when collecting cells from muscle tissue of animals, it is necessary to perform trichotomy, cleaning, and antisepsis of the region of the animal where the biopsy will be performed before collection, as well as subsequent inspection of the sampled tissue for signs of contamination [28]. It is recommended that the supplier confirm that the cell line has been tested negative for infectious agents affecting the donor animal and that the biopsy samples and stem cells collected be analyzed to ensure that they are free of diseases for cultured meat production [28].

All cultures must be stored and maintained under aseptic conditions to avoid contamination from cells that may outgrow the culture and spoil a batch. This requires that all laboratory equipment and work surfaces be sterilized and maintained safely during the processing steps associated with cell culture. Consequently, rigorous cleaning/sterilization protocols with rigorous management practices, such as documentation of all cleaning, and adherence to good cell culture practices (GCP) are required [27,50]. Contamination by microorganisms in harvested cells is a hazard, and any vials of propagated cells that are contaminated with microorganisms should be destroyed and the destruction documented. Another potential hazard is contamination by prions, so avoiding using cells from tissues, such as brain, spinal cord, lymphoid tissue, tonsils, appendix, enteric nervous system, and blood, especially from infected animals, is necessary. In the case of detection of prion infection, it is recommended to adopt the same conduct as mentioned for contamination by microorganisms [24,29,38].

Cell line transport, storage time, and temperature are critical for cultured meat. To manage the hazard, shipments of cold chain materials should be shipped with adequate refrigeration to maintain a consistent temperature throughout the shipping cycle, with a safety margin of at least 24 h of delay. For short-term transport of cells, it is recommended to transfer samples to sterile tubes containing Dulbecco’s Modified Eagle Medium (DMEM) with 1% penicillin–streptomycin (PS) [28]. These tubes can then be placed in a cooler at 4 °C with an appropriate cooling agent, such as ice. For long-term transport, the primary approved method is cryopreservation. Transport is accomplished using dry carriers containing absorbent material, which can be filled with liquid nitrogen to sustain temperatures for up to 2 weeks if adequately loaded [28].

In these cases, chemical substances used for cell release and preservation, such as cryoprotectants including dimethyl sulfoxide (DMSO), sorbitol, or inulin, must be quantified and evaluated from a food safety perspective. In the case of DMSO, it may be present in quantities lower than 2 ppm in the final product. Cryoprotectants, such as butanediol, must be used in the minimum quantity necessary to achieve the intended effect, and quantified in the final product for safety assessment [39]. Cryoprotectants, such as formamide and methanol, are not permitted in the final product. In order to avoid contamination with cryoprotectant residues, it is recommended that cell banks be stored in vapor media as an alternative to liquid nitrogen or that alternative cryoprotectants already used safely as food processing aids be used [39]. It is crucial to perform a new test upon arrival of the cells at their destination to detect any potential contamination that may have occurred during the transportation process [25].

#### 3.4.2. Hazards Associated with Cell Line

Although the genetic stability of cells tends to be conserved as an evolutionary trait [24], when selecting starting cell lines, it is essential to consider that unintended and potentially harmful genetic changes may occur during the production process of cultured meat. To this end, genetic stability assays should be used to mitigate this risk by collecting samples of the cells for chromosome analysis and other sequencing studies to understand the degree of change that has occurred and its safety for human health [47].

The stem and progenitor cells used to produce cultured meat are very complex and differentiated. Predicting specific potential hazards from using different cell lines is challenging, as each company will likely work with its own cell line, which presents specific hazards. In general, it is necessary to ensure sufficient control over the process to correctly differentiate and mature the cells to obtain a final product comparable to traditional meat. Even if the cells may have differentiated correctly, the maturation of the cells may be incomplete and not present the composition that would be expected for a natural muscle fiber. This may not be a problem depending on the target nutritional profile, but this information must be available, and no false claims must be made in the marketing of the product [50].

In addition to incomplete maturation, there is a potential risk that cells will not differentiate properly, producing cells of inadequate quality or a different cell type (e.g., carcinogenic cells). Therefore, quality assurance is required to ensure that the desired differentiation and maturation occur and that the cells that make up the final product are verified (e.g., using biomarkers to track differentiation or genetic techniques). Cells can be tested using assays for potential tumorigenicity, genetic stability, and chromosomal analyses to monitor and mitigate genetic drift. It is also important to highlight the need to characterize cell lines, assess the safety of genetically modified cells and their products, and identify potential expression of toxins or allergens according to regulatory aspects [25,47]. Although experts state that the sequence of events required for a pluripotent or immortalized cell to survive and form tumors after consumption is unlikely, the need to carry out tests to confirm the absence of tumorigenicity of cultured meat through product sampling and risk communication is highlighted [25,48].

Another aspect to be considered concerning the cell line is the expression of new toxins or a change in the expression of toxins as a result of genomic instability (e.g., rearrangements), genetic or phenotypic instability (e.g., variability due to cell division or mycoplasma contamination), or induction by physical or chemical stimuli during cell culture. To be characterized as a potential hazard, these substances would have to be characterized as causing harm to the consumer, but not to the cell culture [25]. This risk can be managed by developing a scientific understanding of the genetic components relevant to food safety, considering the differences and varieties of species and/or cells being used. Risk assessment of these components is necessary to establish the safe level of the remaining components in the final product, considering the exposure levels compared to the reference counterpart to determine whether they affect food safety. In addition, genetic and phenotypic stability assessment can be performed by molecular techniques (e.g., karyotyping) that can indicate the rates of spontaneous genetic changes that may be relevant to food safety [25].

The use of cell lines from animals that are not commonly consumed in that country may also pose a risk, increasing the risk of transmission of new diseases and viruses (if the cell lines are not adequately tested and authentic), as well as potential allergenic reactions to new proteins [50]. In addition, an additional risk to be assessed regarding the cell line is the use of human cells for the production of cultured meat, which may result in so-called “victimless cannibalism”. In this case, the regulatory body must prohibit and monitor the use of human cells to produce cultured meat for human consumption [62].

#### 3.4.3. Hazards Related to Cell Culture Infections

Cell cultures can be infected with other cells, bacteria, yeast, fungi, viruses, prions, endotoxins, and mycoplasma, which can impact culture performance or spoil the culture [42,50]. Cell lines can be infected with cells or microorganisms unintentionally introduced into the culture system or through cross-contamination. This can impact culture performance or even spoil it. Bacterial, fungal, or yeast contamination (or infection) can be caused when a rapidly growing microorganism dominates the culture, uses up its resources, and outcompetes the selected cell line. The infection can be visible under a microscope or to the naked eye due to turbidity and/or identified by the unpleasant odor that some of them give off. This type of contamination can occur through contaminated reagents, contaminated air, water baths, equipment, and cell cultures that are not sanitized or do not follow cleaning protocols, good laboratory practices (GLP), and good manufacturing practices (GMP). In cell culture, these infections are usually controlled with growth-promoting antibiotics and fungicides [50].

In the event of infection, rapid action must be taken to protect neighboring cultures and bioreactors and prevent the infection from spreading. Strict quality control and critical control point measures must be adopted to eliminate infected cells, ensuring clean and sterile work surfaces. Corrective actions must also be taken, such as discarding the infected batch, training operators, sanitizing equipment, and improving monitoring [24,42].

When mycoplasma infections occur (gram-negative bacteria that do not have a cell wall and do not respond to the effects of many antibiotics), unlike the others, they do not cause turbidity in the culture, but they impact the culture by slowing cell growth and altering growth rates, cellular metabolism, physiology, and causing chromosomal changes [50,84]. Therefore, cell lines should be screened regularly for mycoplasma infections using DNA staining, fluorescent staining, and PCR detection, as they are more difficult to detect under the microscope. Although there are many types of mycoplasma and most mycoplasma infections are harmless to humans, some of them can cause infections in humans (e.g., *Mycoplasma genitalium*, *Mycoplasma hominis*, *Mycoplasma pneumoniae*), in addition to the negative effect on the production of cultured meat, demonstrating the need for detection and control measures [50,85]. In the case of mycoplasma infection, the cells should be disposed of by autoclaving, incineration, or disinfection and disposal. It is possible to decontaminate the infected cell line, but this is a more challenging practice and must ensure adequate cell development and product safety with complete mycoplasma disinfection [50,85,86].

In addition to the hazards mentioned, there may be the presence of endotoxins, which are toxic substances originating from the cell membrane of some gram-negative bacteria, such as *E. coli*, *Salmonella*, *Shigella*, *Pseudomonas*, *Neisseria*, and *Haemophilus*. Microbial toxins may be present due to microbiological contamination during any production stage and are characterized by hydrophobic and heat-stable lipopolysaccharides that can contaminate laboratory equipment and become present in the cell culture [25,49]. Due to their hydrophobic nature, they can adhere to contaminated laboratory equipment or utensils (e.g., plastic stirrers) and be transferred to the cell culture. The most common sources of contamination are impure water, laboratory equipment/utensils, culture media, reagents, and serum. To be characterized as a hazard, microorganisms capable of producing a toxin or the toxin itself must be present in the biopsy specimen, human operators, water, air, equipment, ingredients, or packaging materials that come into contact with the cell culture or food product [25].

Microbial toxins pose a risk to human consumption if they are not degraded, washed, or detected and eliminated during food processing, are resistant to food preparation, and are present in the final product at a dangerous level to consumers [25,49]. Due to their heat-stable nature, they may not be destroyed using heat sterilization processes. Endotoxins can produce variability in cell culture, but it is not yet certain that they pose a risk in cultured meat production, as they have not been empirically assessed [25,50]. Controls for this risk should include adopting good practices, such as responsible use of antimicrobials and quality control of raw materials. In the case of cell supply, this risk can be controlled by having access to animal health information. In addition, in cases where there is limited health information about the source animal, toxin detection tests should be performed. When toxins are detected, their dilution factor should be calculated and verified at acceptable safe levels [25].

Viral infection of cultures is difficult to detect because viruses cannot be seen with the naked eye or with standard laboratory microscopes. Viral infection of cell cultures can originate from the donor organisms originally used to extract the cell source or from other animal-derived components, such as serum media, feeder cells, and other derived components used in cultured meat production. Viral infection can also originate from other common contamination routes in cell cultures where there is poor adherence to good practice processes and failure to maintain adequate aseptic techniques [50,87].

It is not yet clear whether cultured meats infected with viruses pose a significant risk to human health, but it is suggested that the risk will be lower than or equivalent to that posed by consuming conventional meat [50,88]. Regarding the spread of viruses to humans from cultured meat, it is known that most viruses are host-specific, limiting their ability to contaminate cell lines. However, they can mutate and infect different hosts. However, there may be a risk that an unknown virus harbored in a host species could be transferred from the cell culture to humans, requiring careful detection and disinfection. If the viruses detected are only cytopathic (morphological), they may not impact on the cell culture. Procedures to remove or inactivate viruses are expensive and characterized by pH alteration by providing an acidic or basic environment or through nano or microfilters [42,50].

There is also contamination of cell cultures by other types of cells due to the use of different types of cell lines. This can occur due to several factors, but mainly due to failure to adopt good manufacturing and laboratory practices, such as poor equipment maintenance, inadequate cleaning of equipment and utensils, incorrect storage of cells, incorrect use of equipment, working with multiple cell lines in the same area, use of the wrong cells, and incorrect labeling of cells, among others. Good manufacturing practices must be followed to ensure that cell lines do not mix and are correctly identified to avoid the hazard arising from contamination by other cells. In addition, identity testing must be performed to authenticate cell lines using PCR, cell line karyotyping, short tandem repeat gene sequencing, or isozyme analysis. In addition, cell lines purchased must come from reliable cell banks that can provide certificates of cell line authenticity [25,50].

It is estimated that cultured meat products will have a better microbiological stability or safety profile than conventional meat due to the potentially sterile conditions during production (with a lower microbiological load in the product at the beginning), leading to a lower risk of product deterioration. Despite this hypothesis, it is necessary to assess microbiological stability and safety and determine the final product’s shelf life [50]. Quality control tests for cultured meat must be validated and documented following applicable national requirements and/or established international standards. Initially, the limits and techniques appropriate to microbiological aspects used for conventional meat can be used to detect and identify microorganisms that may affect the safety of cultured meat [25,89,90]. However, there is a need for adaptation regarding potentially contaminating microorganisms for cultured meat, since the contaminating microorganisms of conventional meat are predominantly of enteric origin and are unlikely to be found in the culture for cultured meat [24].

#### 3.4.4. Hazards Related to Contamination by Components Used in Cell Cultures

During cell culture, culture medium and serum are necessary for cells to proliferate and differentiate. Other materials come into contact with cells, such as microcarriers and scaffold structures, all of which can represent potential hazards [42,47]. The hazards associated with components used in cell cultures can be chemical and physical and, in addition to direct contamination, they can come from physicochemical transformations of food components used in production. This hazard can be characterized by toxic heavy metals, pesticides, herbicides, fungicides, persistent organic pollutants (e.g., perfluoroalkyl substances, polycyclic aromatic hydrocarbons, and dioxins), the residual presence of substances that can migrate from production processes to food contact materials, such as decomposition products of components and integral or residual structural materials [25,81]. Potential sources of this type of contamination are air, water, ingredients, equipment, cleaning products, and packaging materials. If any of these contaminants are present in the final product at levels that would be harmful for human consumption, it poses a hazard [25].

Chemical hazards may be associated with food additives, residues, veterinary drugs, and components of the nutrient medium (cell growth modulators and the structural components of the nutrient medium) introduced into the nutrient medium and migrating into the final product. It is advisable to assess the risk to public health posed by these factors to establish additional requirements for the safety of cultured meat. Since in vitro meat is a new food product and is not yet produced on an industrial scale, assessing the risk to consumer health of the physicochemical transformation of components that are not typical of “traditional” meat (structure, residual components of the nutrient medium, etc.) is difficult, and the presence of this type of hazard should be considered [81].

Some cell isolation, proliferation, and differentiation protocols use potentially toxic chemicals for human consumption. Examples include those used to differentiate preadipocytes into adipocytes, dexamethasone (a steroid), indomethacin, toxic xanthine, and IBMX25, which are not allowed in food products [50,81]. Chemical additives in cultured meat are considered to be chemicals intentionally introduced during production and are present in the final product. Because they are present in the product that will be consumed, the primary strategy to control the hazard involves using only substances for which the toxicity profile is known. There is evidence that the expected level of exposure is safe through authorizations, listings, approvals, or notifications from the appropriate agencies. The general principles of safety assessment of food additives are widely accepted and are applied to all uses of substances added to food. In the case of new additives, it is necessary to evaluate the product’s safety for consumption and regulate its application in food [25,50].

Chemical residues in cultured meat are those chemicals intentionally introduced during production (e.g., antibiotics, antifungals, surfactants, pH buffers, and pH indicators, among others), but without the intention of being present in the final product. The use of antibiotics and fungicides, such as penicillin, streptomycin, and gentamicin, in cell culture characterizes a potential chemical hazard due to their potential residues in the final product. Although culture can occur without using these products, they are likely used to prevent culture infection at some stage of the laboratory meat cultivation process. Therefore, the risk of exposure to antibiotic and fungicide residues needs to be assessed for human consumption and is characterized as a potential hazard when they are not degraded, metabolized, or washed during the procurement, production, and collection of cells, food processing, and reach the final product in a concentration that exceeds the safe level [25,50].

This hazard can be controlled by limiting the use of antimicrobials in the production of cultured meat; eliminating or reducing the need for antimicrobials during culture through aseptic practices; adopting washing procedures to remove or reduce the concentration of antimicrobial agents; and performing analysis and defining specifications that determine the maximum residual level allowed in the harvested cellular material. It should be noted that this hazard is not exclusive to cell-based foods, but also in the use of antimicrobials in conventional food production, including integration into packaging materials, direct addition to foods, and use as a feed additive or veterinary drug in livestock and aquaculture [25].

Some substances used to balance the culture medium, such as those used to control pH (buffers and indicators) and foam formation (antifoams and surfactants), can be used in the different phases of the culture and are not usually metabolized by the cells, generating potential concerns about food safety resulting from the presence of residues of these substances in the harvested cellular material or in the final product at levels that are dangerous to the consumer. For this hazard to occur, a hazardous chemical or additive must be used in the production of cultured meat; the substance must not be degraded, metabolized or washed during the stages of obtaining, producing, and harvesting cells and processing the cultured meat, reaching the final product at a concentration that exceeds the maximum safe level of that residue for human consumption. As with other chemical hazards, control must include the use of minimum levels of substances necessary to achieve the desired technical effect in the culture; use of adequate and validated washes in the harvesting of cells; assessment of potential consumer exposure based on process analysis or analytical data on appropriate safety profiles concerning anticipated consumer exposure [42]. Many of these substances are commonly used in conventional food processing applications (e.g., sodium hydroxide, phosphoric acid, stearic acid, polyethylene glycol, ascorbic acid, and lecithin), so information on safe use levels is available [25].

The hazards arising from physicochemical transformations of food components arise from interactions between components present in the product and other substances that lead to modifications in the compound structure, generating the undesirable occurrence of reactive species and other compounds that are harmful to health. These transformations may occur during food processing after cell harvesting (e.g., smoking, heat treatment, and chemical treatment) or during the sterilization of inputs during production (e.g., irradiation) in substances that are sensitive to the method used. To characterize the hazard, the transformed compounds must be detected in the final product and present in the final product at a dangerous level for human consumption [25,81]. The potential hazard can be controlled by carrying out a safety assessment of the final product, including the analysis of the chemical transformation of the primary food components, which must be tested on new ingredients with no history of safe use for use in food. Ingredient evaluation (in silico and in vitro) should be used to track the reactivity of the components. If it is identified that the transformations pose a risk to human consumption, there is a need for control measures to prevent the occurrence of these dangerous transformations. It is important to note that cell-based foods may include ingredients and inputs not commonly found in conventional meat (e.g., scaffolds and residues) that may result in new physicochemical transformations that should be considered in the risk assessment of cultured meat processing techniques [25].

The sources of culture medium and serum are considered challenging, as they may have residues in the final product. In particular, the use of fetal bovine serum (FBS) is strongly discouraged by experts, both for ethical reasons and because it may present some potential hazards [47]. The use of new serum-free alternatives that are not yet used in food production must be evaluated for their permanence in the product and their safety for human consumption, and this product must undergo an authorization process. Therefore, residual testing based on toxicological standards is recommended to evaluate the residues that remain in the final product and their safety for human consumption [47,50].

The most commonly used components to control cell proliferation, differentiation, and maturation are growth hormones, steroids, sodium benzoate, collagen, xanthan gum, mannitol, cochineal, omega-3 fatty acids, morphogenic proteins, transforming growth factor, Zfp423 protein transcription factors, myokines, adipokines, cytokines, interleukin-1, interleukin-6, interleukin-10, hepatocyte growth factor and tumor necrosis factor-alpha, MYOD, MRF4, TGF1, testosterone, progesterone, and muscle-specific regulatory factors. These compounds can be absorbed by the cell or associated with the cell wall and transferred to the final product. Therefore, investigation is needed into how these compounds can bioaccumulate in the final product and how they are removed or allowed at an acceptable exposure level [24,25,35,37].

In addition to those mentioned above, there are also several compounds used in the formation of scaffolds for the production of cultured meat, such as collagen, extracellular matrix proteins, chitosan, polyethylene, polystyrene, epoxy, poly(glycolic acid), poly(lactic-co-glycolic acid), polylactic acid and poly(N-isopropyl acrylamide), gelatin, fibrin, hyaluronic acid, agar, dextran, alginate, Cytodex, RGD linkers, RHO-associated protein kinase inhibitors, and fibronectin [50,78]. During the production process, it is possible that scaffolds break down, degrade, or leave residues, potentially contaminating the final product. Therefore, it is also necessary to investigate these compounds in the final product and how they are removed or whether they are at an acceptable exposure level. Table 5 presents the materials commonly used in scaffolds, their characteristics, potential hazards, and actions to control the hazard.

The components present in bioreactors or used in their cleaning can pose a risk and are mainly antifoaming agents, anticoagulant agents, diluting agents, thickening agents, cleaning chemicals, sterilization chemicals, NaOH, emulsifiers, surfactants, and bioreactor-specific materials, such as metal and plastic. In addition to representing a physical hazard, plastic can produce toxic particles and inhibitory chemicals in cell lines. In addition, cells can come into contact with metal components of the bioreactor, allowing heavy metals to leach into the final product [50]. Therefore, it is necessary to investigate whether any of these products are present at an unacceptable exposure level in the final product. Furthermore, bioreactors must be rigorously sterilized and cleaned using, for example, heated steam, sodium hydroxide, and 70% ethanol solution. Residual chemicals left over from cleaning can be transferred to the final product if not appropriately managed [50].

A study conducted in the United States examined the effects of exposure to microplastics, represented by fluorescent polyethylene microspheres (10–45 µm) on cellular performance, including cell proliferation, cell viability, gene expression, and differentiation processes critical for the production of cultured meat [51]. The results indicated, as a potential hazard, the presence of microplastic in cells of origin from marine animals prone to microplastic bioaccumulation, showing the need to investigate the cells of origin for microplastic detection. Furthermore, it highlighted the widespread presence of microplastics in the laboratory, ingredients, and during the production of cultured meat, increasing the risk of unintentional contamination by microplastic, requiring the detection of microplastic in the final product, and evaluation of the potential impacts on human health in the long term [51].

Microplastics are small particles of matter derived from the degradation of plastics found in food, water, and/or air. Microplastics pose a potential hazard in food or can interact with other components, altering their properties and creating a hazard. To pose a health risk, the microplastic must be introduced into the cell culture process or the final product, be undetectable, and be present in the final product at a dangerous level for consumers [25]. To control the hazard of microplastics, good practices must be followed, specifically related to the filtration of source materials and reducing the use of plastics in contact with food [25].

#### 3.4.5. Hazards Related to Nutritional Aspects

The risks related to nutritional aspects are highlighted due to the possibility that the nutritional profile of the final product (cultured meat) may be different from that which it is replacing (conventional meat) [50,91,92,93]. Some nutrients in traditional meat are not present in cultured meat if these components are not added, such as vitamins B12 and D, creatine, and iron, since these products are not created in the muscle cells but transported to them [50]. Furthermore, it is necessary to evaluate whether the quality of the protein provided by cultured meat is similar to that of traditional meat. In the case of different nutritional quality between cultured meat and natural meat, nutrients must be added to present a nutritional profile similar to that of the reference product. In this sense, there is also a need to evaluate whether the added nutrients will present bioavailability similar to that of traditional meat. In the case of a divergence in the nutritional composition concerning its counterpart (traditional meat), the consumer must be informed to enable them to decide whether to consume the product [50,82].

Considering that cultured meat does not have the sensory and nutritional properties consistent with traditional meat, flavorings, colorings, vitamins, and minerals can be added to the culture to mimic these characteristics, and their impact on the chemical composition of cultured meat needs to be evaluated. It is important to highlight that there is a possibility of improving the nutritional quality of cultured meats, such as controlling the quantity and quality of the lipid profile (e.g., adding omega 3, reducing cholesterol, and saturated fat), vitamins, and minerals with possible benefits to the health of the population [50,82]. However, all of this must be evaluated and regulated by competent regulatory bodies.

It is important to note that nutrients used in culture media can also potentially pose a hazard when considered as residues in the final product. Although these are substances frequently found in foods, if one or more of these substances were present in the final product at dangerous levels to the consumer, it would be a concern for food safety. In this case, to pose a hazard, the nutrient used in the culture media must be accumulated and present in the cellular material or in the final product at a level considered dangerous to consumers. To control the hazard, it is recommended to use minimum levels of nutrients in culture media sufficient to achieve the desired cell growth and to monitor cellular parameters during culture, as well as the nutritional quality of the food comparable to its counterpart. It is also necessary to analyze the composition of the cellular material to identify nutrients that are present at a harmful level. Since many of the nutrients used in culture media are present in a wide variety of conventional foods, and there is already information on safe consumption levels for these substances, the established standards can also be used for cultured meat [25].

### 3.5. Safety Assessment Strategies and Actions in Cultured Meat Production

Safety assessment strategies and actions for a new product, such as cultured meat, are based on the principle that the safety of new products is assessed relative to a conventional counterpart with a history of safe use, considering both intended and unintended effects, as hazards can occur inadvertently at any time during the production of cultured meat. Therefore, standardized procedures and practices, such as good laboratory practices, good manufacturing practices, good cell culture practices, and a code of hygienic practices, are prerequisites for the safe production of cultured meat [25,29,38,81].

Any significant differences between the new and conventional products must be assessed from the point of view of potential adverse health effects. To assess the safety of a new product, it is crucial to determine whether the modification could develop or increase pathogenicity, toxicity, or allergenicity. The fundamental strategy is the application of risk and hazard management systems, which are currently mandatory in many countries and mainly include hazard analysis and critical control points (HACCP) and hazard analysis and risk-based preventive controls (HACPR). Thus, the potential hazards at each stage of cultured meat production are pre-estimated, and consequently, the risks are reduced or avoided [29]. To this end, some strategies must be adopted, including routine biochemical evaluation, allergenicity tests, toxicity tests, kinetic tests, and genetic tests [25,29].

#### 3.5.1. Routine Biochemical Evaluation

Although it is still uncertain whether all standards adopted for conventional meat products apply to cultured meat products, these standards potentially provide pre-established benchmarks for cultured meat. Therefore, harmful microorganisms, viruses, prions, and mycoplasmas common in cultured meat are anticipated to be monitored similarly to traditional meat. Unintentional residues and the formation of harmful byproducts can occur due to the addition of various ingredients and exposure to processes, materials, and equipment in cultured meat production. Cultured meat production should adhere to the same maximum levels of metals, natural toxins, agricultural or veterinary chemicals, and environmental contaminants as in traditional meat products previously stipulated, and their risk assessment can be carried out according to previously established guidelines [25,82,83,94].

For example, antibiotics can be avoided in the animals from which the cells are collected and by using a sterile environment. However, if used, they must be detected, characterized, and quantified in cultured meat to determine safety. It is worth noting that many antibiotics currently authorized for use in animals are also approved for humans, which minimizes concerns related to residues derived from them. However, in addition to the previously authorized components, some new materials used in cultured meat may pose challenges to their safety assessment. Given this, the detection of new components needs to be specifically developed based on conventional analytical methods (chromatographic, mass spectrometric, immunological methods, etc.) and existing bioassays [24,25,82].

To assess chemical contaminants, it is necessary to identify and quantify the levels of chemical contaminants specific to the food produced. Contaminants may be naturally present in the food before the application of the new process or may be introduced as a result of the application of the new process. It is important to compare the levels of chemical contaminants in the new food with those levels typically found in the food product prepared by accepted traditional processes [25].

The detailed composition (e.g., macro and micronutrients, bioactive compounds, toxins, and allergens) of cultured meat products needs to be determined and quantified through laboratory testing and compared to their counterparts [95,96]. Novel products with equivalent composition and functional aspects to already regulated foods should be as safe as their counterparts. However, equivalence does not guarantee the absolute safety of the novel food, it only provides greater assurance that it is potentially as safe as conventional food, reinforcing the need for product testing [25]. The composition of cultured meat products differs from that of traditional meat products due to compositional differences between species, breeds, sex, and cuts of conventional meat, as well as the presence of novel components in the cultured meat. Therefore, even if the main component of cultured meat complies with the different traditional meat products established by regulatory agencies, a risk assessment for the novel components is necessary [25,27].

In assessing dietary exposure to substances with no history of use as foods intended for use as foods, it is important to determine the following: (i) how much of the food is likely to be consumed and how frequently, and what role it is likely to play in the diet (e.g., as a source of protein, etc.); (ii) the potential impact of this food on dietary nutrient intake, highlighting information on the nutritional composition of the food; (iii) if there are any antinutrients, toxins, contaminants, or novel substances in the food, to estimate the potential exposure to these substances [24,25,28,82].

#### 3.5.2. Allergenicity Tests

Allergenicity assessment is necessary to prevent susceptible individuals’ unexpected and/or unavoidable exposure to food allergens. For foods with no history of safe use, there is the potential for one or more proteins to cross-react with known food allergens or lead to the development of hypersensitivity [36]. A protein-based product, such as cultured meat, presents a higher risk of allergenicity since most allergens are characterized by glycosylated proteins (although this is not the only possibility for allergy). Therefore, if a novel protein is found in cultured meat products (different from that found in conventional meats), there is the potential for increasing the allergenicity of the product [25,82].

Therefore, there is a need to determine the amino acid sequence, and its structure should be compared with known allergenic proteins registered in databases using bioinformatics analysis. If homologous sequences or structural similarities with known allergens are observed, additional allergenicity tests are required, such as in vitro digestive stability test, allergenicity assay using cell lines and/or rodents, human serum IgE test, skin prick test, and controlled food challenge study, among others [24,25,38].

Clinical studies on allergenicity may be considered unethical due to the potential severity of the resulting clinical manifestations. Therefore, they should only be performed after obtaining the results of in vitro allergenicity tests and after authorization by the Ethics Committee. In addition, attention should be paid to the influence of the processing method on the putative allergenic potential during the consumption of cultured meat. If the identified allergens are detected in the cultured meat, information about the allergens must be provided on the labels to guide consumers appropriately [25,36].

#### 3.5.3. Toxicity Test

Assessment of toxicological aspects is necessary to determine whether any unknown hazards may have been introduced during food production. Conventional toxicity studies on the final product may be required, including metabolism, genotoxicity, cytotoxicity, toxicokinetics, chronic toxicity/carcinogenicity, impact on reproductive function, and teratogenicity. When designing appropriate toxicity tests, all relevant materials, processes, and details involved in producing cultured meat should be carefully considered.

The main toxicity tests include genotoxicity, cytotoxicity, and toxicity tolerance tests. Cytotoxicity tests analyze abnormal cell growth, function, morphology, disintegration, and death caused by chemicals. The most relevant are colorimetric tests, fluorometric tests, and luminometric tests. Genotoxicity tests refer to the damage to the DNA or chromosomes of cells, resulting in mutagenic or carcinogenic effects. Therefore, in food, this analysis aims to identify substances that can cause genetic damage to humans, predict potential genotoxic carcinogens, and understand the mechanism of action of chemical carcinogens [25,28].

#### 3.5.4. Kinetic Tests

Kinetic testing in cultured meat is a laboratory method that monitors bioprocess parameters, such as temperature, pH, and cell density, to ensure cell culture quality. It refers to the analysis of absorption, distribution, metabolism, and excretion, which can characterize how nutrients from novel foods are systemically absorbed, distributed throughout the body, and eliminated. Typically, absorption testing (in vitro, in vivo, and ex vivo) and in vitro gastrointestinal metabolism are performed initially. Only compounds that are systemically absorbed, broken down, or metabolized into components that can be absorbed during gastrointestinal metabolism require subsequent ADMET (absorption, distribution, metabolism, excretion, and toxicity) testing or higher-level toxicology studies to generate more extensive data. Kinetic data combined with nutritional data provide references for further toxicity testing. Post-market monitoring through consumer feedback (especially regarding adverse effects) needs to be tracked after a given novel food is allowed to enter the market, which can be used as clinical data to conduct a comprehensive safety and risk assessment of that novel food [39].

#### 3.5.5. Process Detailing and Monitoring

The entire cultured meat production process must be detailed and monitored. Facilities where cultured meat is produced must have a food safety plan that includes hazard analysis and risk-based preventive controls to minimize or prevent identified hazards [39,49]. In addition, a qualified preventive controls professional must be available to perform or supervise the activities. A food safety plan (FSP) consists of the primary documents, including product description, production flowsheet, process description, hazard analysis, preventive controls (allergen preventive controls, sanitation preventive controls, and temperature preventive controls), verifications (facility sanitation verification), and implementation, in a food safety system of preventive controls that provides a systematic approach to identifying food safety hazards that must be controlled to prevent or minimize the likelihood of foodborne illness or injury [39].

#### 3.5.6. Genetic Testing

Genetic testing can provide a comprehensive understanding of cellular characteristics, such as genetic stability, through the manufacturing process. Genetic testing can identify the cell source and determine the number of passages within the laboratory where no significant mutations or loss of function are detected. Genetic modifications refer to cells modified by modern genetic engineering techniques or unintentional mutations occurring in producing cultured meat. To mitigate the hazard related to modifications, it is necessary to describe the genetic modification with information on the modification method used, description and characterization of the potentially delivered genetic material, if applicable, including the source, identity, and expected function in the organism. It is also necessary to detail the manipulations or modifications to the introduced, intermediate, and recipient genetic material, and to provide information on the added, inserted, deleted, or modified DNA, including the characterization of all genetic components, including marker genes, regulatory elements, and others that affect the function of the DNA, the size and identity, the location and orientation of the sequence in the vector/final construct, and the function in the organism. Finally, it is necessary to characterize the genetic modification through information on the DNA insertions in the genome, as well as substances expressed in the modified cell [24,25,39].

#### 3.5.7. Actions to Support Safety Assessment

A study by regulatory experts highlighted key actions to support the safety assessment of cultured meat products [48]. These actions are based on:

(i) Process understanding: aims to distinguish standard and innovative manufacturing approaches across the industry and identify and characterize potential input components of culture media, structural materials, cell lines, etc.;
(ii) Product understanding: aims to identify common adventitious agents that may be introduced during manufacturing and be present in the final product; establish the shelf life of cultured meat; evaluate the genetic drift of cell lines under production conditions; establish data on residue levels and potential metabolites of inputs; evaluate the potential for accumulation vs. dilution of chemical or biological contaminants or toxicants; identify new allergens and toxins, including endogenous and exogenous substances introduced during manufacturing; evaluate the composition and compare with their counterparts; evaluate the natural variation in the composition of cultured and conventional meat products;
(iii) Development of safety methods and approaches: aims to adapt and validate microbial assessment methods; adopt good practices and HACCP appropriate for cultured meat; establish safe limits for maximum cell passage during production; develop and validate methods to assess genetic drift; develop approaches for residue risk assessment; expand knowledge about the threshold of toxicological concern with the inclusion of classes of substances relevant to cultured meat (i.e., bioactive molecules); develop methods to identify new toxins or allergens, including approaches for comparison with conventional products; assess the stability of inputs and metabolites; determine relevant parameters to characterize cell lines; establish criteria for conventional comparators and identify compositional parameters that support safety assessment or nutritional evaluation; develop relevant comparative assessment approaches and acceptable ranges of nutritional and compositional parameters;
(iv) Publicizing and making available data and methods: aims to establish publicly available parameter databases for composition, common inputs, and microbiological parameters; publish peer-reviewed safety research in the public domain; develop standard safety test methods that provide industry-wide benchmarks [48].

### 3.6. Regulations and Guidance Documents on Cultured Meat

As cultured meat is an innovative food product, regulations and oversight structures are essential to ensure the quality and safety of the product for human consumption [28]. Due to the innovation in the production process, cultured meat is considered a novel food and must follow the general rules established for this type of product [36]. The entry of new foods into the market must undergo prior authorization based on a risk assessment of safety for human consumption regulated in several countries to ensure consumer health protection [25,27,97].

In this context, the global regulatory landscape for cell-based foods has been changing. After the Singapore Food and Agriculture Organization (SFA) approved the first cell-cultured product in 2020, the United States authorized two cell-cultured products in 2022 and 2023, followed by the Israeli government approving the first cell-based beef product in 2024. EFSA and Food Standards Australia New Zealand (FSANZ) are also establishing and reviewing the issues [98]. Regulations, reports, and guidance documents from regulatory agencies in different regions of the world that are relevant to the production or commercialization of cultured meat are described by region.

#### 3.6.1. Singapore

Regulatory approvals for cultured meat are ongoing and are gradually being rolled out in some countries. Singapore was the first country to grant regulatory approval for cell-based meat products in 2020. The Singapore Food Agency (SFA) granted marketing approval for a lab-grown chicken meat product [22]. According to the SFA, safety should be a primary consideration when companies develop foods. As such, alternative proteins with no history of consumption as food will only be allowed for sale once they have been deemed safe for consumption [22]. In 2024, the SFA updated the Regulation for Novel Foods and Novel Food Ingredients, which regulates the marketing of foods with no history of safe use [99]. In this regard, producers interested in bringing new products (such as cultured meat) to market are required to submit safety assessments to the SFA so that the agency can evaluate the data [99]. Regarding cultured meat, the safety of the product is reviewed by the SFA at three different levels, focusing on the following: (i) production process (cell lines, culture media, reagents, toxicology, etc.); (ii) process and controls ensured (e.g., contaminants, adherence to good safety, and hygiene practices); (iii) final product that must meet the standards established by national food regulations [22,99]. According to the SFA, labeling must indicate that the cultured meat is derived from cell culture so that the customer is aware of what he or she is purchasing or consuming [27,100]. The SFA has established an approach to safely assess biological substances used in cultured meat production media [22].

#### 3.6.2. United States

In 2022, the United States Food and Drug Administration (FDA) granted pre-market approval for cultured chicken meat products, indicating that the product is safe for human consumption [101,102]. In Singapore and the United States, restaurants already offer cultured meat with consumer consent [82]. In the US, the FDA and the USDA created a joint regulatory approach in 2018, followed by establishing a joint agreement in 2019, to address the regulation of cultured meat [103]. The FDA oversees cell collection, cell banking, cell growth, and differentiation, while the USDA oversees the cell culture harvest stage onward (production and labeling of products). The agreement reflects that foods produced from animal cells cultured for human consumption are regulated based on the animal species used as the original source of the cultured cells. Other applications of this cell-cultured food production technology that are not covered by the formal agreement are within the jurisdiction of the FDA. In this regard, the FDA regulates the processing of foods, as well as the foods themselves, made from cultured cells of animals not regulated by the USDA (e.g., non-siluriformes seafood and wild game). As outlined in the formal agreement, the FDA’s approach to regulating products derived from cultured cells involves a thorough premarket consultation process and inspections of registrations and facilities, as applicable [103].

The premarket review process assesses the safety of foods produced using cultured cells before they enter the market. It allows developers to work with the FDA on a product-by-product basis and inform them of issues they must consider for producing safe foods that do not violate federal requirements. In this process, the FDA evaluates the production process and cultured cell material, including the establishment of cell lines and cell banks, manufacturing controls, and all components and inputs to support innovation in food technologies while always keeping the production of safe food for human consumption as a priority [103].

The FDA shall conduct routine inspections of cell banks and facilities where cells are cultured, differentiated, and collected to ensure that potential risks are managed and that biological material emerging from the culture process is safe and unadulterated. The FDA shall ensure compliance with applicable requirements for facility registration, good manufacturing practices, and other food safety regulations [102]. The FDA shall also ensure that labeling cell-cultured products derived from animal species that are not subject to USDA jurisdiction is truthful and not misleading, consistent with the General Principles for Product Labeling and Claims [102,104]. The FDA has established an inventory of completed premarket inquiries for foods made from cultured animal cells that sets forth the following information: (i) a description of the food and species of origin; (ii) a file number that the FDA assigned to the premarket inquiry; (iii) the sponsor’s final submission in the inquiry explaining its basis for concluding that the cultured animal cell material is safe for use as human food; (iv) a letter that the FDA sent in response to the sponsor after the premarket consultation; (v) scientific memorandum that documents the FDA’s evaluation of the sponsor’s final submission, as well as presents the list of completed premarket consultations [105].

In 2022, the FDA published Cell Culture Consultation (CCC) memorandum 000001, Cultured *Gallus gallus* cell material, which evaluated the food subject of CCC 000001 submitted by the company GOOD Meat [106] and Cell Culture Consultation (CCC) Memorandum 000002, Cultured *Gallus gallus* cell material from UPSIDE [107]. For the purposes of the consultation, food was defined as the cellular material at harvest, composed of cultured *Gallus gallus* cells, with fibroblast characteristics, in the form of cellular biomass, as produced by the manufacturing method described in the document [106,107]. The companies describe the overall production process involving establishing a cell bank that provides a standardized source of cells for food production and a cell culture food production process, including proliferation or multiplication of the cells and harvesting or collection of the cellular material for subsequent conventional food processing. The company declares that a food safety and quality system is in place during production and provides information on the following programs and measures that will be used in its production facilities, including: (i) current good manufacturing practices program; (ii) development of a food safety and hazard analysis and risk-based preventive controls (HACCP) plan, including preventive measures and corrective actions for prevention and mitigation of biological, chemical, and physical hazards; (iii) in-process checks and controls of key process parameters; (iv) documentation of the implementation and/or validation of process controls, sanitation, and environmental controls and supply chain controls; (v) product release system involving quality assurance review for incoming raw materials, intermediate products, and finished products; (vi) batch record review; and (vii) traceability of raw materials and finished products [106,107]. In GOOD Meat’s document, the company states that its cell culture production process follows internal standard operating procedures (SOPs) and is performed by authorized and trained personnel that include crisis management, document management, employee training, general cleaning procedures, and recall procedures, and that cells are grown in a controlled environment that utilizes high-efficiency particulate air (HEPA) filters and differential pressure to maintain air quality [106]. All of the safety and quality systems and procedures outlined in the document are important for the safe production of cultured meat. The documents state that no processes that have been identified would result in any contamination by any substance or microorganism that would adulterate the cultured meat and that the foods mentioned are as safe as their counterparts produced by other methods [106,107].

The Food Safety and Inspection Service (FSIS) inspection has program personnel regarding their roles and responsibilities with respect to inspection and verification activities at establishments that harvest or process cell-cultured meat or poultry food products for human consumption. The directive also provides instructions on how to request registrations or other information from the FDA related to the production of cell-cultured meat or poultry food products in the United States, as well as review and approve product labels so they can be marketed [108]. It also states that foreign countries may not export cell-cultured meat to the United States unless the FSIS has determined that they have a regulatory food safety inspection system equivalent to that of the United States for cell-cultured meat production. When the FSIS determines a country is equivalent for such products, it will list the country as eligible to export cell-cultured meat to the United States [108]. Cultured meat is subject to the same FSIS import and export regulations and policies as its food product counterparts [108].

The FSIS is revising the directive to reflect the updated Memorandum of Understanding (MOU) between the FSIS and the FDA and to provide guidance to inspection program (IPP) personnel at establishments that collect cells for cell-cultured meat and poultry food products [109]. The document states that district offices (DOS) must report to the FSIS headquarters liaison, the appropriate FDA field office, and the FDA headquarters liaison for the following situations: (i) the discovery of food involved in outbreaks of foodborne illness, injury, or adverse health consequences; (ii) the discovery of food that is adulterated or misbranded such that there is a reasonable probability that use of or exposure to such products will cause serious adverse health consequences; (iii) a condition or processing failure that is likely to result in contamination of food, leading to outbreaks of foodborne illness or serious adverse health consequences; (iv) significant findings at the facility of unsanitary conditions, such as rodent infestation; (v) any positive results from a microbiological or other sampling project. These results must include microbial characteristics (e.g., serotype, whole genome sequence, antimicrobial resistance profile), where applicable, and other information related to pathogen categorization and tracking, such as findings of harborage or cross-contamination; (vi) all for-cause sampling results, including positive and negative results; (vii) the initiation of a recall; (viii) reports of adulteration or threats of adulteration; (ix) a food handler diagnosed as having a communicable disease likely to result in food contamination or foodborne illness outbreaks (e.g., hepatitis); (x) convictions of a dual jurisdiction establishment (DJE), or any officer or key employee of a DJE, for any felony or more than one misdemeanor involving the DJE or any food prepared or packaged at the DJE; and (xi) FSIS action to withhold the mark of inspection or to suspend or withdraw the grant of inspection.

In 2024, the USDA-FSIS published a notice (FSIS Notice 28-24) that provides instructions regarding FSIS Notice 31-23 (Cell-Cultivated Meat and Poultry Food Product Sampling Program, published in 2023 [110]) and updated it to indicate that, after the initial 10 samples for a specific cell line or production method have been collected, the FSIS will collect samples on a quarterly basis. Additionally, FSIS Notice 28-24 provides instructions to inspection program (IPP) personnel on how to collect cell-cultured meat products and submit samples to the FSIS laboratory for microbiological, chemical residue, speciation, and pathology testing. It also instructs Inspection, Investigation, and Analysis Officers (EIAOs) on collecting environmental and food contact surface samples from cultured meat production sites and submitting them to the FSIS laboratory [111]. This document describes that the FDA and the USDA-FSIS jointly oversee cultured meat production, with the USDA-FSIS responsible for the process during harvest and post-harvest production of cell-cultured meat and poultry products. Therefore, it establishes that the USDA-FSIS will conduct sampling analysis of cultured meat and food products in the following circumstances: (i) the samples of raw products will be tested for enumeration of aerobic microorganisms, *Salmonella*, chemical residues, speciation, and pathology (microscopic anatomy and histological examination); (ii) environmental swabs and food contact surface (FCS) samples in establishments that produce raw products will be tested for enumeration of aerobic microorganisms and *Salmonella*; (iii) samples of ready-to-eat products will be tested for aerobic microorganisms, *Listeria monocytogenes* (Lm), chemical residues, speciation, and pathology (microscopic anatomy and histological examination); and (iv) environmental samples and swabs from food contact surfaces in establishments producing ready-to-eat products will be tested for aerobic microorganisms, *Listeria monocytogenes* [111].

The USDA-FSIS also published a 2024 rule regarding voluntary labeling of FSIS-regulated products with U.S. origin claims, stating that labels on FSIS-regulated cell-cultured meat and poultry products are not currently eligible for generic approval under the Agency’s prior label approval system. Therefore, the FSIS will review all labels and claims on these products before marketing to ensure they are truthful. Voluntary “Product of the USA” and “Made in the USA” label claims will be permitted on cell-cultured products only if all cell preparation and processing steps occur in the United States [112].

#### 3.6.3. Canada

In Canada, the revised 2022 Novel Food Guidelines cover novel foods derived from plant or microbial sources and highlight that safety assessment criteria for novel foods derived from animals are under development [113]. In Canada, novel foods and food ingredients are considered novel under the Food and Drug Regulations and require a pre-market safety assessment to demonstrate that they are safe before they are marketed. In the case of cultured meat, it is highlighted that, although the guidelines are still under development, the basic requirements will be as follows: (i) conduct the safety assessment of cell-cultured foods on a case-by-case basis (ideally within 410 calendar days of receiving a pre-market notification of a novel food); (ii) consider all specific aspects of a product, including its development and manufacturing processes; and (iii) consider the potential for harmful chemical and microbial contaminants, allergens, toxins, and nutritional quality. It also highlights that cellular agriculture products will follow generic rules on food hygiene and safety, including good manufacturing practices (GMP), hazard analysis and critical control points (HACCP), and the general prohibitions of the Food and Drugs Act, which include the prohibition on selling a food manufactured under unsanitary conditions, or containing a poisonous or harmful substance [114]. Health Canada’s information document on cultured meat advises that, for an overview of the scientific, safety and other considerations related to cellular agriculture, the FAO document should be consulted [25,114].

#### 3.6.4. European Union

EU countries do not yet have specific regulations for cultured meats. However, the EU Novel Foods Regulation (EU 2015/2283) ensures that novel foods are safe for human consumption in Europe [115,116]. According to a document published by the EFSA in 2024, under current EU legislation, foods and food ingredients derived from cell cultures require pre-market authorization under different sectoral legislation, for which the EFSA’s scientific advice on their safety may be necessary. Therefore, the EFSA’s risk assessment approaches must be updated with new scientific and technical innovations to protect human health and consumer interests [116].

The European Commission (EC) has asked the EFSA to update the scientific guidance for preparing applications for the authorization of novel foods, previously developed under Regulation (EU) 2015/2283 on novel foods [43,115]. The aim is to define the scientific information the applicant requires to demonstrate the safety of the novel food, including those using cultured cells. The document includes foods from cell culture or tissue culture derived from animals, plants, microorganisms, fungi, or algae [43], and establishes that implementing food safety management systems in place to produce novel food must encompass procedures based on HACCP principles [43].

The scientific guidance for foods produced from animal cells indicates the following needs: (i) indicate the identity of the source organism, including information to attest that the primary cells and tissues used for the preparation of the novel food meet the established inspection requirements; (ii) when using established cell lines, indicate the genetic and phenotypic identity and stability of the cells; (iii) when using primary cells, indicate the site of the biopsy or source material, type(s) of cell(s) isolated, genetic and phenotypic identity of the cells; (iv) present information to attest the absence of any risks of infectivity from viruses or other zoonotic agents, e.g., tests for viruses (species-specific viruses), tests for prions in the case of limited health information on source animals; and (v) present information on whether the cells or tissues originating from a non-genetically modified animal have been genetically modified after the biopsy [43].

The guidance further states that if primary cells are used, information on the source, purification steps, cell isolation, cell selection, cell subculture, absence of pathogens, and microbial contaminants must be provided. If cells from established cell lines are used, information must be provided on the source, cell line preparation, cell banking process, as well as the passage number of the aliquot of cells used. Any changes made to the cells used (e.g., selection, differentiation, immortalization, adaptations, reprogramming) and the link of such changes to the production of potentially hazardous substances must be described. All processes applied for treating, extracting, screening, and selecting cell lines or tissues must be provided in detail, including all chemicals and biological materials used and impurities that may result from their use [43].

It further determines that the genetic stability of cells throughout the production process should be investigated by comparing the starting material (i.e., initially selected cells from biopsy/cell line) and cells at different stages of the production process. Changes in morphology, differentiation markers, and other phenotypic characteristics of cells at the beginning and end of the production process should also be investigated and described. Information on compliance with good cell culture practices and with the relevant applicable standards on the derivation and characterization of cell substrates used to produce biotechnological/biological products is required. The safety of growth factors of microbial origin, such as recombinant proteins, vitamins, and amino acids used in the production of cell-derived foods, will be assessed to establish the safety of the novel food, taking into account the scientific requirements for taxonomic and risk identification of microorganisms intentionally used in the food chain following the relevant EFSA guidance documents [43].

The requirements set out refer to the description of the novel food, production process, composition data, specifications, proposed uses, and anticipated intake levels of the novel food. In addition, the necessary information on its source, absorption, distribution, metabolism, excretion, toxicological information, nutritional information, and allergenicity must also be described. According to the guidance, the applicant must provide general considerations on how the information supports the safety of the novel food under the proposed conditions of use; where potential health hazards are identified, they must be discussed concerning the anticipated intake of the novel food and the proposed target populations. Based on the information provided by the applicant, EFSA will assess the safety of the novel food under the proposed conditions of use [43].

Despite progress in the EU, the Italian government has proposed a specific law for 2023 banning foods made, isolated, or produced from cell cultures or tissue cultures derived from animals, which includes cultured meat [117]. In the approved version, the production, use, sale, import, distribution, and promotion of cultured meat (defined as “synthetic meat”) will be prohibited, considering the possible risks to the health of consumers, and to the livelihood of the Italian agricultural sector [117].

In the United Kingdom, the *Food Standards Agency* (FSA) is working on developing new food regulations for cultured meat production processes. At the time of this review, there was no authorization to market cultured meat in the United Kingdom. However, a document has already been prepared regarding the production process and identifying hazards in cultured meat [50]. This document establishes the potential components that can be used in cultured meat and pose a hazard, namely:

(i) Components to control cell proliferation, differentiation, and maturation: growth hormones, steroids, sodium benzoate, collagen powder, xanthan gum, mannitol, cochineal, omega-3 fatty acids, bone morphogenic proteins, transforming growth factor β, zinc finger protein transcription factors Zfp423, myokines, adipokines, cytokines, interleukin-1, interleukin-6, interleukin-10, hepatocyte growth factor and tumor necrosis factor alpha, MYOD, MRF4, TGF1, testosterone, progesterone, and muscle-specific regulatory factors.
(ii) Growth medium components and components added to keep cells alive and nourish them: glucose, amino acids, vitamins, minerals, serum medium, serum-free medium, growth factors, binding proteins, adhesion factors, hormones, oxygen-carrying trace elements, modified hemoglobin, perfluorochemicals, feral bovine serum, HS, L-glutamine, E2, TBA, TBA-E2, inorganic salts, buffer systems, carbohydrates, antibiotics, Dulbecco’s Modified Eagle’s Medium (DMEM), Glutamax C, inhibitors, and activators of cellular pathways, e.g., p38 inhibitor SB203580, poloxamers.
(iii) Scaffolding materials used to support cell growth: collagen, coating materials, extracellular matrix proteins, laminin, cellulose, chitosan, polyethylene, polystyrene and epoxy, poly(glycolic acid), poly(lactic-glycolic acid), polylactic acid and poly(N-isopropyl acrylamide), gelatin, fibrin, Matrigel and elastin, such as hyaluronic acid, chitosan, agar, dextran, or alginate, Cytodex, RGD linking groups, RHO-associated protein kinase inhibitors, and fibronectin.
(iv) Bioreactor components and cleaning chemicals: antifoaming agents, anticoagulant agents, diluting agents, thickening agents, cleaning chemicals, sterilization chemicals, NaOH, defoamers, emulsifiers, surfactants, and the material of the bioreactor itself, e.g., metal, plastic.

#### 3.6.5. FAO and WHO Members

Although they are not considered regions, different countries are members of the FAO and WHO. Therefore, the regulatory, guidance, or information documents related to them were addressed in this topic, as some documents are joint.

In 2022, the FAO held the first meeting on cell-based foods in collaboration with the Israeli Ministry of Health, where a group of developers and researchers in the field of cell-cultured food production shared information on the products they had developed and discussed issues relevant to food safety [98]. This led to the stakeholder meeting organized by the FAO together with the China National Center for Food Safety Risk Assessment (CFSA) in November 2023 [118].

In 2023, the FAO and WHO published a joint document to provide a bibliographical synthesis of relevant terminological issues, principles of cell-based food production processes, and the global panorama of regulatory frameworks for cell-based food production [25]. The FAO and WHO also published a document in 2023 that summarizes information from the previous one [25], highlighting the following nine main points related to cultured meat: (i) characterization; (ii) nomenclature; (iii) cell-based foods are no longer considered products of the future; (iv) aspects related to food safety; (v) sustainability aspects; (vi) action of food safety authorities; (vii) international scenario related to cultured meat; (viii) action of regulatory bodies to minimize consumer concerns regarding food safety; and (ix) measures that competent authorities need to consider [119].

The FAO and WHO published a report in 2024 [98] that provided an overview of the state of development of cell-based food technologies and precision fermentation (a process that uses microorganisms to produce specific target products through controlled production systems with growth media and bioreactors). In this document, it was possible to see examples of how to assess the safety of products using these technologies since a growing number of countries are reviewing regulatory applications [98]. According to the document, most regulatory frameworks in different regions require pre-market approval for cell-based food products. Since a food safety assessment is one of the elements for approval, if companies plan to commercialize a given product, they need to be able to prepare an extensive dossier containing a comprehensive food safety assessment of their product [98]. This document also highlights the attention to six main categories of food safety issues as follows: (i) the genetic stability of cells/cell lines; (ii) microbiological risks related to cell lines; (iii) exposure to substances used in the production process; (iv) toxicity and allergenicity for the general population; (v) risks of post-harvest microbiological contamination; and (vi) chemical contamination/residue levels [98].

Later in 2024, the FAO and WHO published a report of the meeting that discussed cell-based food safety aspects in the context of the “Near East” region (a transcontinental region around the Eastern Mediterranean that encompasses parts of Western Asia, the Balkans, and North Africa) [120]. The report indicates that Qatar, Saudi Arabia, the United Arab Emirates, Egypt, the Islamic Republic of Iran, Oman, Sudan, and Yemen are in the early stages of regulatory discussions on cell-based foods and many planned to consult with key stakeholders in cultured meat production so that a proper assessment could be carried out to decide what regulatory actions were needed [120].

#### 3.6.6. Israel

In 2024, the Israeli Ministry of Health approved the commercialization of cultured beef, provided that the products undergo safety assessments, meet specific labeling requirements, and obtain a license from the Ministry of Health. Because cultured meat introduces new food manufacturing technologies and this product has no prior history of safe human consumption, it will fall under the category of “novel foods”. The Ministry of Health will review various aspects that will ensure the promotion of this field in Israel, while also protecting Israeli public health. The specific food regulations in this country are in Hebrew, and are not presented in English, which made it impossible to evaluate the documents for this review [121].

#### 3.6.7. Australia and New Zealand

Food Standards Australia New Zealand (FSANZ) is the agency that develops the standards for regulating the use of food ingredients, additives, and processing aids in Australia and New Zealand, and is responsible for some labeling requirements for packaged and unpackaged foods, including specific mandatory warnings or advisory labels. FSANZ published an information report on cultured meat in 2023, which mentions that there are still no permissions or requirements in its Food Standards Code for this type of food. However, it highlights that cultured meats would follow the same standards as those in the Code for other foods and require pre-market approval. Depending on the composition of the cell-based meats, these standards may include the following: (i) novel foods—foods with no history of traditional human consumption in Australia and New Zealand; (ii) processing aids—substances used to produce food but which do not play any technological role in the final food for sale; (iii) food additives—substances that play a technological role in the final food for sale; (iv) foods produced using gene technology; (v) vitamins and minerals; (vi) labeling indicating the true nature of the food; (vii) definition of cell-based meat; and (viii) food safety requirements [122].

#### 3.6.8. Brazil

In Brazil, there is still no specific regulation regarding the production of cultivated meat. The bodies responsible for analyzing the approval of new foods are the Department of Inspection of Products of Animal Origin of the Ministry of Agriculture, Livestock and Food Supply (DIPOA/MAPA) and the General Management of Food of the National Health Surveillance Agency (GGALI/ANVISA), which will probably be responsible for analyzing the requests for approval of cultivated meat products.

A regulatory study on cultured meat conducted in Brazil suggests that the regulator should focus more on the protocol for submitting new products than on minimum and maximum parameters related to the finished product and indicating the analytical methods for evaluating the product and inputs used. This protocol for submission to the regulatory agent should contain detailed information about the production process, production control system, and identified risks, ingredients, inputs, and adjuvants used, product characteristics, risk analysis of its use as food, as well as the name of the product, intended use, and recommended consumption [24].

Due to the lack of specific regulations in Brazil for cultured meat, it is recommended that those applied to their counterparts and other general regulations (such as those referring to new products) be used as a basis for the regulation and authorization for cultured meat, since food regulations in Brazil are well established. Resolution RDC No. 839 of the National Health Surveillance Agency (ANVISA), published in 2023, provides proof of safety and authorization of the use of new foods and new ingredients, establishing standards for registration, including those produced from cell culture [97]. This resolution includes products that “consist of cell cultures or tissue cultures or have been produced from such cultures” and highlights the requirement that new foods and new ingredients that are constituted, isolated, or produced from cell or tissue cultures must be identified based on the following criteria: biological origin of the culture containing the scientific name, according to the most current and scientifically recognized nomenclature; organ, tissue, or part of the organism of origin; laboratory or collection of culture of origin; identity of the cells; cells or tissue substrate used; and type of culture [97]. Furthermore, these products must be characterized based on the following information: description of methods used for screening and selecting cells or tissues; description of modifications made to cell lines and their relationship with the expression of substances that may result in a risk to human health; information on the preparation and maintenance of cell lines; list of ingredients that make up the culture media and culture conditions used and information on the purity and genetic stability of the cell or tissue culture during the manufacturing process [97].

As mentioned, although there is no specific regulation regarding cultured meat in Brazil, this product is addressed in Resolution RDC No. 839 of the National Health Surveillance Agency (ANVISA) published in 2023 [97]. Considering the potential hazards related to cultured meat found in this review, national documents can be used as a basis to guide actions and regulations associated with this product.

In general, Resolution–RDC No. 275/2002 of ANVISA, which provides for the Technical Regulation of Standardized Operating Procedures applied to Food Producing/Processing Establishments and the Checklist of Good Manufacturing Practices in Food Producing/Processing Establishments [123] and Ordinance No. 46/1998 of the Ministry of Agriculture, which aims to establish the system of hazard analysis and critical control points–HACCP to be implemented in industries of animal products, [124] can be applied to the production of cultured meat, except for the need for updating due to the novelty of the product.

In the context of cultured meat production, one of the risks raised was the presence of medicines (or their residues) and contaminants present in the final product. In Brazil, ANVISA Resolutions RDC No. 623/2022 and 722/2022 provide for the tolerance limits for foreign matter in food, the general principles for their establishment and the methods of analysis for conformity assessment purposes and the maximum tolerated limits (MTL) of contaminants in food, the general principles for their establishment and the methods of analysis for conformity assessment purposes, respectively [125,126]. ANVISA Resolution–RDC No. 730/2022 assesses the risk to human health of veterinary medicines, the maximum residue limits (MRL) of veterinary drugs in food of animal origin and the methods of analysis for conformity assessment purposes [127]. Furthermore, ANVISA’s Normative Instructions–IN No. 241/2023, IN No. 162/2022 establish the acceptable daily intake, the acute reference dose and the maximum residue limits for active pharmaceutical ingredients of veterinary drugs in foods of animal origin [128,129] and ANVISA’s Normative Instruction IN No. 160/2022 establishes the maximum tolerated limits of contaminants in food [130]. These criteria can be used as a basis for establishing standards for cultured meat to minimize hazards related to potential hazards from chemical and physical contaminants.

Furthermore, to minimize potential chemical hazards, ANVISA’s RDC No. 272/2019 [131] can be used to determine the additives permitted in cultured meat production since it establishes the food additives authorized for use in meat and meat products. In 2024, the Ministry of Agriculture and Livestock established the Official Methods for Analysis of Products of Animal Origin—Chemical Methods [89], which can support the analyses to be carried out with cultured meat products.

Regarding the hazards of microbiological origin, Normative Instruction–IN No. 60 establishes the lists of microbiological standards for food [132], which can be a guiding document for establishing standards for cultured meat. For the analysis of products of animal origin concerning microbiological aspects, the Ministry of Agriculture and Livestock established, in 2024, the Manual of Official Methods for Analysis of Products of Animal Origin—Microbiological Methods [89], which can be used as a basis for microbiological analyses regarding cultured meat.

In the final phase of preparing the cultured meat product, the Questions and Answers Manual on Labeling Allergenic Foods published by ANVISA, in 2024, provides guidance on the labeling of foods that come into contact with materials that are made with allergen derivatives, minimizing the hazards related to allergens [133]. Within the scope of informing consumers about the products that will be consumed, Resolution–RDC No. 843/2024 provides for the regularization of foods and packaging under the jurisdiction of the National Health Surveillance System [134] and Resolution–RDC No. 727/2022 provides for the labeling of packaged foods [135], which can guide the criteria to be applied to cultured meats, but which needs to be updated to include specific aspects related to the new developments regarding this product. In 2024, the Ministry of Agriculture and Livestock published the Technical Regulations for the Identity and Quality of Products of Animal Origin—Meat and its derivatives [90], which can be used as a standard for cultivated meat, except for the specificities that may arise from the novelty of the product. Another guiding document for cultivated meat, considering its counterpart, is the Manual of Inspection and Monitoring Procedures for meat and meat products in establishments registered under Federal Inspection (SIF) published by the Ministry of Agriculture and Livestock in 2022 [136].

### 3.7. Other Critical Points: Main Bottlenecks in Cultured Meat Production Around the World

The choice of suitable cell lines is a critical challenge [137]. Lineages must be efficient in proliferation and differentiation to form tissues similar to conventional meat. Genetic stability and mutation control are essential to guarantee safety and consistency in production, avoiding the risk of unwanted mutations and potential tumorigenicity [138].

Culture media provide nutrients necessary for cell growth. For large-scale production, there is a need for fetal bovine serum-free media that are safe, economical, and sustainable. The high cost and the need for specific components make it difficult to achieve economic viability on a large scale, as well as questions about the safety of the growth factors used [139].

Scaffolds act as three-dimensional support for cells to grow and organize themselves, replicating the texture of flesh. These supports need to be biodegradable, edible, and safe. Producing effective scaffolds that replace natural collagen is still challenging, as they must provide the desired structure without compromising safety or taste [140].

The limited availability of bioreactors capable of supporting large-scale production is an obstacle. Current bioreactors were developed for other types of cell cultivation, and difficulties are faced in maintaining the sterility and stability of large volumes. Investment in specialized equipment is needed to meet growing demand and guarantee the quality of the end product [141]. In addition, although cultured meat has been considered environmentally friendlier than conventional meat (mainly due to high greenhouse gas–GHG-emission, use of fertilizers on feed-crop fields, carbon gas emissions, and water use in traditional livestock), limited data is available to address this topic. Cultured meat is suggested to have the potential to reduce the environmental impacts related to meat production, specifically regarding GHG emission, land, and water use [142]. A study showed that cultured meat reduces 7–45% of energy use, 78–96% of GHG emissions, 99% of land use, and 82–96% of water use compared to conventional meat production [87]. Despite the improvements in animal welfare, the systems designed to produce cultured meat must be more efficient, avoiding emissions associated with the production process. However, cultured meat production might come with increasing energy use during the process due to all industrial processes replacing natural ones [143,144,145]. Tools such as life-cycle assessment (LCA) and others should be used to evaluate the environmental impacts of cultured meat production, allowing the evaluation of the impacts related to all the stages of a product’s life cycle, including the extraction of raw materials, processing, manufacturing, distribution, and use [142,143]. The assessment of the environmental impacts of cultured meat depends on data provided by relevant actors of cultured meat production. Consumer acceptance and the social and economic consequences of cultured meat might also compromise the environment and sustainability, and it must be evaluated by the government, society, and all actors involved in cultured meat production and consumption [142,143,144,145].

These bottlenecks represent critical areas for advances in cultured meat production, and the development of appropriate technologies and regulations to overcome these challenges is essential.

### 3.8. Limitation

This study has several limitations that should be acknowledged. First, the absence of a standardized testing framework for cultured meat presents a challenge in ensuring consistent safety and quality assessments across different products. Additionally, the findings are reliant on existing regulatory frameworks, which vary significantly between regions, and are still in development, potentially limiting the applicability of the conclusions.

The novelty of cultured meat introduces uncertainties, as unforeseen safety risks and concerns may not yet be identified in the current literature. While this study highlights documented hazards, such as genetic stability, microbiological risks, and chemical contamination, it may not fully capture emerging or undocumented hazards associated with this innovative food technology.

Furthermore, the regional variability in regulatory approaches may affect the generalizability of the findings and recommendations, as different regions adopt diverse policies and guidelines. Finally, the limited exploration of shelf life, spoilage mechanisms, and long-term safety implications leaves critical gaps that require further investigation to ensure the viability and safety of cultured meat products over time.

## 4. Conclusions

The development and commercialization of cultured meat require clear and up-to-date regulation and oversight. Therefore, it is essential to establish a strong regulatory framework to ensure the safety, quality, and accurate labeling of cultured meat products. In addition to chemical, biological, and physical hazards (including genetic and residue aspects), it is important to consider the following elements in the risk assessment of cultured meat: tissue biopsy, cell banking, possible harmful byproducts, storage, allergenicity, product stability, and production scale-up. Another risk raised in the documents assessed is that the novelty of the product and its process may introduce new, as yet unknown, safety concerns. In this sense, the regulatory documents recommend attention to the following key points of food safety related to cultured meat: (i) the genetic stability of the cells/cell lines; (ii) microbiological risks related to the cell lines; (iii) exposure to the substances used in the production process; (iv) toxicity and allergenicity of the product or component for the population; (v) risks of post-harvest microbiological contamination; (vi) chemical contamination/residue levels; in some regions, they include (vii) nutritional aspects/risks. The novelty of cultured meat brings potential unknown hazards, and their impacts cannot yet be assessed. More studies on the uncertainty factors need to be developed and strengthened throughout cultured meat’s production, commercialization and consumption processes. New technological tools or long-term monitoring programs should be developed and explored, in addition to existing testing methods to minimize potential hazards.

Currently, there is no single approach to testing different types of cultured meat. However, effective risk and safety assessment strategies should be adopted. Most of the documents assessed in this review consider using an appropriate HACCP food safety system to ensure the safety of cultured meats, associated with good practices in all spheres of cultured meat production. It was also found that the regulatory framework for cultured meat in different regions may evolve differently but should follow the basic principles of safety for human consumption to enable the development of a safe and nutritious product. Overall, the documents indicate that cultured meat production is safe, provided that adequate monitoring systems are put in place and that many of the safety criteria can be guided by the requirements established for its counterpart (traditional meat). However, further studies must assess the environmental impacts involved in cultured meat production and consumption since it also impacts on the sustainability and safety of the planet.

Some points were highlighted as necessary: (i) characterize which foodborne pathogens can grow or survive in the final product during storage and distribution; (ii) evaluate the behavior of spoilage microorganisms in the product, in the shelf life and safety of cultured meat; (iii) verify the potential cross-contamination during packaging and the growth potential of foodborne pathogens throughout the shelf life of the product; (iv) determine the shelf life of cultured meat products considering the conditions of formulation, processing, storage, distribution, and marketing; (v) identify the limiting factor for the shelf life for cultured meat products; (vi) establish strategies or validate procedures necessary to reduce or remove chemical residues from the final product; (vii) evaluate whether cell culture inputs not commonly used in conventional food production present allergenic/mutagenic/carcinogenic/toxic potential and identify their acceptable levels in the final product; (viii) evaluate whether microorganism toxins are present in the final product; and (ix) investigate differences and similarities in the transformation of muscle into meat between cultured meat and conventional meat and the possible implications for safety and quality.

Government and regulatory agency documents point to a trend toward applying the concept of novel foods and implementing pre-market assessment to ensure food safety for cultured meat. These aspects can form the basis for developing international regulations and promoting the production and consumption of alternative meats. In general, regulations, guidelines, and authorizations regarding cultured meat should include the following: (i) pre-market consultation to evaluate materials and production processes; (ii) supervision of cell collection and the quality of cell banks; (iii) supervision of the production process until harvest; (iv) ensuring compliance with the requirements established by the country’s regulatory bodies; (v) issuing additional regulations or guidelines when necessary to ensure that biological materials leaving the culture process are safe; (vi) inspections and surveillance aimed at the safety of cell banks and culture facilities; (vii) inspections of establishments where cells are harvested, processed, packaged, or labeled to ensure that the resulting products are safe, unadulterated, wholesome, and appropriately labeled; and (viii) establishing additional requirements to ensure the safety and accurate labeling of cultured meat products.

Future research on new cultivation technologies to produce cultured meat, the construction of more accurate risk assessment models and environmental impacts assessment should be performed in light of current research’s shortcomings and the industry’s development trend to promote in-depth research in this field.

## Figures and Tables

**Table 1:** Characterization of studies.

Author and Reference	Year	Country	Type of Study	Objective	Hazards/Critical Points	Hazard Control/Recommendations
Goswami et al. [34]	2024	India, United States, Netherlands	Narrative review	Address the biomaterials, production processes, tissue engineering approaches, processing, quality, safety, regulatory, and social aspects of cell-cultured seafood.	(1) Use of antibiotics, such as penicillin, streptomycin, ampicillin, gentamicin, and kanamycin, in fish culture for microbial decontamination. (2) High ammonia production in the cell proliferation and differentiation phases. (3) Presence of allergens, such as parvalbumins, tropomyosin, arginine kinase, and myosin light chains.	(1) – (2) Use of glutamine substitutes, including α-ketoglutarate (αKG), glutamate (Glt), and pyruvate (Pyr) had a positive impact on cell proliferation and differentiation by reducing the rate of ammonia production. (3) Selective cultivation of specific cell types (e.g., myogenic and adipogenic cells) to avoid allergenic components; or genetic modifications, such as the use of RNA interference (RNAi) techniques to eliminate causative genes; or incorporation of additives, such as creatine or ethylenediaminetetraacetic acid (EDTA), into the cell culture medium to modulate the expression of allergens (e.g., parvalbumin), thereby reducing allergenicity.
Huang et al. [35]	2024	Singapore Netherlands and United States	Scoping review	Review the scientific literature on growth factors commonly used in the production of cultured meat.	(1) Presence of growth factors in the final product.	(1) Establishing the safety of using growth factors in cultured meat requires analyzing residual levels of growth factors in the final cultured meat product before and after cooking, compared to the slaughtered meat counterpart.
Lanzoni et al. [36]	2024	Italy	Narrative review	Provide an overview of the current legislative, food safety, technical, and economic challenges of cultured meat.	(1) Contamination of the cell derived from the animal of origin. (2) Contamination by veterinary drugs. (3) Cryoprotectants. (4) Genetic instability with formation of cancer cells. (5) Abnormal nutrient accumulation. (6) Use of fetal bovine serum. (7) Hormones. (8) Antibiotics. (9) Chemicals used in the medium (antifoams, pH buffers, culture media stabilizers) and accidental introduction of microplastics from water, equipment, and the external environment. (10) Physical-chemical transformations that can generate changes that are harmful to the consumer’s health. (11) Allergens.	(1) Rigorous inspection of source animals and biopsied cells/tissues for signs of infection. (2) Perform tests to quantify veterinary drugs in both the cell line and the final product; consult the health data of the source animals at the time of tissue biopsy. (3) Cryoprotectants must be eliminated or diluted to very low concentrations and combined with washing of the final product. (4) Assessment confirming absence of risk. (5) Use of minimum levels of nutrients that still allow cell growth; constant monitoring of cellular parameters (e.g., viability and morphology) as indicators of cell damage; chemical analysis of the final product to identify the nutrients present, compared to the maximum safe levels related to intake are already known for traditional foods. (6) Use of animal-free culture media. (7) Use of these substances in minimum concentrations that still allow the desired effect to be achieved, the use of product washing steps and, finally, the implementation of safety and quality control measures. (8) Replacement of antibiotics with natural or synthetic antimicrobial peptides, lysins, bacteriocins, hydrolyzed peptides, and biological extracts, which do not constitute a stress factor or create drug resistance; document and record the use of antibiotics or a substitute, the type and concentration, increasing controls for human health safety. (9) Quantify the levels of these chemical contaminants at all stages, right through to the final product. (10) Evaluate and test the physical-chemical transformations of the ingredients included in the formation of the final product. (11) Selecting substances from non-allergenic sources, using minimum levels of these substances, quantifying potential residues in the final product, and assessing potential consumer exposure.
Lee et al. [37]	2024	Korea	Narrative review	Compile information on the history, development and current level of technology, industrialization status, and future prospects of the cultured meat industry.	(1) Exposure to contaminants in the collection of animal muscle tissue cells. (2) Contamination of stem cells by chemicals or recombinant proteins. (3) Growth factors and hormones present in fetal bovine serum (FBS) used in culture media. (4) Substances used in the manufacture of cultured meat include extracellular matrix, basic culture fluid, and serum not yet authorized for use in food.	(1) Use of induced pluripotent stem cells or embryonic stem cells; use of antibiotics and antifungals in the cell storage process with assessment of potential levels of residues retained in the cells. (2) Assess the safety and legality of using chemicals or recombinant proteins in food. (3) Use of proven safe FBS for the production of cultured meat or use of serum-free media. Conduct carcinogenicity and toxicity testing when these materials are used. (4) Assess whether these materials should be replaced by edible materials or considered waste substances, based on research into the development of alternatives or safety verification of existing substances.
Macedo et al. [28]	2024	Brazil	Narrative review	Explore the importance of quality and risk control in cultured meat production, elucidating how management at each stage impacts both product safety and quality.	(1) Obtaining and characterizing cells: (i) use of raw materials with no proven history of safe consumption; (ii) adoption of animal cell culture components and carriers that have no history of safe use in the cultured meat production process; (iii) the new procedure involved in the production of cultured meat, which includes genetic modifications to alter the expression levels of certain nutrients; and (iv) the consumption of transgenic cell lines. (2) Cell bank: presence of bacteria and fungi; cellular alteration with tumorigenic potential; reagents or materials used that have genotoxic potential; reagents present in the final product; elements such as toxins and residues of products used in cell culture (medium, antibiotic, dimethyl sulfoxide, and animal proteins); presence of cryoprotectants in the final product. (3) Bioreactor: Rapidly growing bacteria, yeasts, and fungi can contaminate meat culture bioreactors through contaminated broth components, air filtration failures, poor cleaning, and incomplete sterilization of equipment, piping, and fittings, or during handling operations when strict aseptic techniques are not followed. (4) Final product: pH (bacterial growth and putrefaction of cultured meat).	(1) Assess the health status of the animal; document any veterinary medications administered to the animal, such as antibiotics (including dates of administration and the established withdrawal period for each medication); and identify any diseases that could potentially affect the safety of the resulting cultured meat product; animals eligible for biopsy must be in a veterinarian-certified healthy condition after evaluation, similar to the existing procedure for slaughter animals for traditional meat production; apply advanced laboratory techniques for in-depth analysis of biopsy samples and stem cells collected, ensuring a disease-free baseline in cultured meat production; donor tissues and/or blood may contain pathogens, such as prions that are typically located in specific tissues, such as the brain, spinal cord, lymphoid tissue, tonsils, appendix, enteric nervous system, and blood from infected animals. Therefore, it is advisable to avoid the use of these tissues; perform shaving, cleaning, and antisepsis of the region of the animal where the biopsy will be performed prior to collection, and subsequent inspection of the sampled tissue for signs of contamination. Transportation and storage: Shipments of materials subject to cold chain management must be shipped with adequate refrigeration to maintain temperature throughout the shipping cycle, accommodating at least a 24-h delay in the expected arrival time. This implies that the critical risk factor for cell transport is temperature. For short-term cell transport, a recommended approach involves transferring samples to sterile tubes containing Dulbecco’s Modified Eagle Medium (DMEM) with 1% penicillin–streptomycin (PS). These tubes can then be placed in a cooler at 4 °C with a suitable cooling agent, such as ice. Dry ice may also be used without the need for medium. For long-term transport, the primary approved method is cryopreservation performed using dry carriers containing absorbent material, which can be filled with liquid nitrogen to sustain vapor phase temperatures for up to 2 weeks if properly loaded. Evaluate factors such as container strength, pressure resistance, temperature controls, choice of refrigeration material (liquid nitrogen or dry ice), and the heat transfer capability of the container material. Assessment of cells for residues upon arrival at the new destination to detect any potential contamination that may have occurred during the transport process. Establish a protocol for cell bank stability, including a table to record test results. Cell bank reports should be fully documented and up to date. (2) Monitor each step of the cultured meat manufacturing process to track potential sources of hazard and contamination. Quality controls at the cell culture stage include cell characterization, cell viability, and cell differentiation, as well as safety tests, such as microbiological, mycoplasma detection, endotoxin, genomic stability, and tumorigenicity. Regardless of the cell type, whether primary, modified, or immortalized, the quality controls are the same. Perform tests for the detection of microorganisms and endotoxins; assess chromosomal instability during cell culture; assess genotoxicity; determine the maximum amount of reagent that can be found in the product; assess impurities related to the cell manufacturing process must be carried out through risk management based on a guideline that defines the detection limits for the presence of impurities in the product, and the best available methods and analyses to assess the presence of impurities; assess the probability of the presence of cryoprotectants in the final product. (3) Even if the microorganism is identified and its susceptibility to antimicrobials is known, the use of chemicals may not be optimal, and it is advisable to discard the batch to avoid potential risks to consumers. In addition, to prevent the spread of microbial contamination, it is essential to protect nearby bioreactors; mycoplasma contamination is not detected by microscopic inspections, so DNA-based methods must be implemented in quality control protocols. Upon detection of contamination, the entire batch should be discarded. (4) Control of the pH of the final product (below 6.0). (5) Legislation regarding cultured meat must cover the entire production chain, from the selection of cell donor animals to the packaged, ready-to-eat product.
Manning [38]	2024	United Kingdom	Narrative review	Analyze existing literature to (i) identify materials that can be used in the cultured meat process; (ii) explore potential biological and chemical food safety issues; (iii) identify food safety risks; and (iv) position a responsible innovation framework that can be utilized to mitigate food safety concerns with specific emphasis on cultured meat.	(1) Allergenicity of materials used in scaffolding (e.g., wheat, soy, albumin, peanut, chitosan). (2) Presence of mycoplasma in cell culture. (3) Presence of endotoxins. (4) Presence of cyanotoxins (e.g., microcystins, saxitoxins, and cylindrospermopsin). (5) Presence of viruses in fetal bovine serum used in culture media. (6) Presence of prions. (7) Potential for formation of polycyclic aromatic hydrocarbons and *N*-nitrosamines if nitrates and nitrites are added to cultured meats as preservatives. (8) Potential for the formation of biogenic amines (such as histamine, tyramine and putrescine) produced in meat due to its high amino acid content, as a result of bacterial activity causing amino acid decarboxylation and other biochemical reactions. (9) Presence of cryoprotectants such as inulin, dimethyl sulfoxide (DMSO), and sorbitol. (10) Use of antibiotics in culture media (e.g., penicillin, streptomycin, amphotericin, and gentamicin) that can be carried over to the final product if there is no control. (11) Presence of unintended metabolites formed during the cultured meat production process. (12) Presence of heavy metals from amino acids and algae materials (e.g., mercury). (13) High levels of iodine when using algae-derived materials.	(1) Assessment of allergenic potential. (2) Use of antibiotics, such as quinolones, macrolides, and tetracyclines, which can pose a risk to public health. In this case, to avoid the use of these antibiotics, the use of UV radiation to eliminate mycoplasma is recommended. (3) Adopt appropriate equipment purification and sterilization procedures and materials testing programs to reduce the risk of endotoxin contamination. (4) Evaluation of water and algae derivatives used in the production of cultured meat for the presence of cyanotoxins. Use of UV irradiation to reduce cyanotoxin levels. (5) Testing of fetal bovine serum for the presence of the following viruses: orbivirus, bovine adenovirus, bovine parvovirus, bovine respiratory syncytial virus, bovine viral diarrhea virus (BVD), rabies virus, and reovirus 3. Discard the material if these viruses are detected. (6) Assess the presence of prions in the material and discard the material if detected. (7) Regulate the addition of nitrates and nitrites and verify the safety of compounds formed from their addition. (8) Assess the presence of biogenic amines in the final product and its safety for human consumption. (9) Quantify and limit the amount of inulin, dimethyl sulfoxide (DMSO), and sorbitol to permitted standards. (10) Identification of the presence of antibiotics and their metabolites in the final product. (11) Conducting safety screening of amino acids used in cultured meat production and assessing the risk of unintended metabolites that are harmful in fermentation-related biotechnology processes. (12) Analysis of the material used for the presence of heavy metals and evaluation of the contact surface to prevent the migration of heavy metals. (13) Quantify the iodine present in the final product and assess its safety for human consumption.
Ovissipour et al. [39]	2024	United States	Hypothetical case study	Develop a plan for the safe production of lab-grown fish meat.	(1) Improper packaging materials. (2) Contamination of receiving cell bank cells. (3) Cryoprotectant dimethyl sulfoxide (DMSO). (4) Cryoprotectants formamide and methanol. (5) Butanediol cryoprotectant. (6) Potentially allergenic compounds. (7) Contamination by Mycoplasma, *Salmonella* or *E. coli*. (8) Contamination by foreign materials (physical). (9) Intentional contamination. (10) High ammonia levels. (11) Growth of spore-forming bacteria (post-processing). (12) Presence of allergens. (13) Presence of fish parasites.	(1) Receiving packaging materials: Inspecting packaging materials for signs of inappropriate odor, damage, or contamination; checking labels to ensure all details are correct; certification by the testing provider indicating that the materials are safe for food contact; storing packaging materials covered and in a segregated area, away from the rest of the plant and any allergenic materials or chemicals; temperature control of stored materials. (2) Obtain cells from healthy animals that are confirmed to be free of potential toxins (mercury, ciguatera toxin, and harmful algal toxins if the cells are from marine organisms); any vials of propagated cells that are contaminated with bacteria or mycoplasma must be destroyed and the destruction documented; if the cells are received from a supplier, request a health certificate and species authentication (DNA barcode). (3) Assess whether the quantity is less than 2 ppm. (4) Not allowed in the final product. (5) Used in the minimum quantity necessary to achieve the intended effect and quantify it in the final product when used. (6) Potentially allergenic compounds should be stored in a separate area. (7) Daily testing for mycoplasmas and microbial testing for other pathogens every two weeks; supply chain control, approved the audit of suppliers. Microbial testing of fish as part of the health certificate sent with each batch of fish cell’s DNA testing; after removal of chemicals in a bioreactor, the media will be UV sanitized, and samples will be collected for sanitation verification; supply chain verification. Temperature control. Microbial testing of hydrogels and bioinks. Temperature control of the cell diffusion process. (8) Removal of material, if present; regular checking of the biofilter to remove any solid materials. (9) Safety and monitoring; safety program and policy; staff training; only authorized personnel have access to the production room and process control room. (10) Collect reactor samples to measure ammonia, nitrite, and nitrate as part of GMPs. (11) Proper labeling and instructions on thawing; use of suitable packaging materials with oxygen transmission rate 10,000 cc/m ^2^/24 h at 24 °C or higher; process control for the temperature of products and transport vehicles with data logger. (12) Detailed description of each step of the cultured meat production process based on a flow diagram; allergen control by label declaration. (13) Freeze and store at room temperature of −20 °C or below for 7 days.
Rao et al. [40]	2024	India	Narrative review		Phase 1 (cell line development): (i) health status of the animal whose cell will be collected; (ii) enzymes/chemicals used for cell release and preservation, such as cryoprotectants like dimethyl sulfoxide (DMSO), sorbitol, inulin. Phase 2 (proliferation): (i) Nutrients and other compounds used in cell proliferation (amino acids, vitamins, minerals, growth factors, antibiotics, etc.). Culture medium requirements are specific to each type of meat cell; (ii) fetal bovine serum used in the culture medium may harbor microbial contaminants and endotoxins. Phase 3 (bioreactor differentiation): (i) Use of growth factors like TGF, FGF, IGF for cell differentiation and oxygen carriers like hemoglobin, myoglobin perflurochemicals. Use of enzymes/chemicals (trypsin, EDTA) during cell harvesting; (ii) nutrient-rich culture medium is vulnerable to contamination by bacteria (mainly mycoplasma); (iii) different types of materials are used for the production of edible and non-edible scaffolds may pose a health risk to the consumer. Phase 4 (product development and post-processing): product contamination due to unhygienic handling practices.	Phase 1: (i) Collection of cells from disease-free animals; (ii) components of the culture medium need to be evaluated from a food safety perspective. Phase 2: (i) components of the culture medium must be evaluated from a food safety perspective; (ii) develop alternatives to fetal bovine serum or use culture medium without fetal bovine serum. Phase 3: (i) growth factors and other components need to be evaluated from a food safety perspective; (ii) development of a standard procedure to prevent bacterial contamination during the production of cultured meat; (iii) edible scaffolds used must not be harmful or cause allergies to consumers; non-edible scaffolds must be biodegradable. Phase 4: Establish standard operating procedures to prevent contamination during handling and production of cultured meat.
Shi et al. [41]	2024	China	Narrative review	Investigate four key aspects to develop a new system for active prevention and control of food chain safety.	Physical, chemical and biological.	(1) Develop effective methods to retrospectively identify and analyze hazards throughout the novel food chain, considering the various categories and sources of these hazards. It is also crucial to develop high-throughput non-targeted screening technology to monitor the dynamic chemical changes of hazards and their interactions with proteins. (2) Develop an artificial intelligence modeling approach and network analysis technology that enables investigation of formation and transformation pathways of non-traditional ingredients, as well as hazard interactions. (3) Track migration patterns of potentially hazardous components in new food raw materials and seek to capture the movements of non-traditional ingredients and hazards to record the extensive data generated by changes in proteins and related substances. (4) Assess the collective impact of hazards across categories in novel foods. Dynamically regulate the emergence of hazards at critical stages of novel food production and effectively prevent the formation of hazardous components.
Sogore et al. [42]	2024	China	Narrative review	Examine the key microbiological and chemical hazards that must be monitored and controlled during the cultured meat manufacturing process.	(1) Microbial contamination in the initial collection of cells from source animals and the environment. (2) Chemical residues during cell proliferation. (3) Waste from scaffolding materials, microcarriers and media components. (4) Contamination from non-sterile conditions, equipment, or worker handling if adequate aseptic conditions are not maintained.	(1) Ensure the microbial safety of donor animals to prevent contamination of cell lines used for cultured meat production. Animals from which stem cells are collected must be raised and slaughtered under sanitary conditions. (2) Identification of residues using techniques such as high-performance liquid chromatography (HPLC), spectroscopy, and immunoassays; elimination of chemical contaminants. (3) Use of edible or biodegradable materials or dissociation of cells from the material. (4) Adoption of good practices and maintenance of aseptic conditions of the environment and materials.
Turck et al. [43]	2024	European Union	Revision	Provide advice on the scientific information required to be submitted by the applicant to demonstrate the safety of novel food.	-	(1) Implementation of food safety management systems in place to produce novel food, encompassing HACCP principles. (2) Indicate the identity of the source organism, including information to certify that the primary cells and tissues used for the preparation of novel food meet established inspection requirements. (3) When using established cell lines: indicate the genetic and phenotypic identity and stability of the cells. (4) When using primary cells, indicate the site of biopsy or source material, type(s) of cell(s) isolated, and genetic and phenotypic identity of the cells. (5) Provide information to attest to the absence of any risk of infection by viruses or other zoonotic agents; perform tests for viruses and prions in the case of limited health information on source animals. (6) Provide information on whether cells or tissues from a non-genetically modified animal were genetically modified after biopsy. (7) To investigate the genetic stability of cells throughout the production process by comparing the starting material (i.e., initially selected cells from biopsy/cell line) and cells at different stages of the production process. (8) Changes in morphology, differentiation markers, and other phenotypic characteristics of cells at the beginning and end of the production process must be investigated and described. (9) Provide information on compliance with good cell culture practices as well as on compliance with relevant applicable standards on the derivation and characterization of cell substrates used for the production of biotechnological/biological products.
Wang et al. [44]	2024	China	Experimental study	To investigate the potential allergenicity and linear epitopes resistant to digestion of fish skin gelatin in cell-cultured meat structures.	(1) Allergenic potential of fish skin gelatin used as a scaffold for cell growth, proliferation, and differentiation in cultured fish meat.	(1) Development of hypoallergenic cellular scaffolds for cultured fish meat production.
Broucke et al. [45]	2023	Belgium	Narrative review	Provide an overview of nutritional, techno-functional, and sensory properties, food safety and legislative/regulatory aspects of cultured meat production.	(1) Selection of cell source: (i) microbial contamination (bacteria, viruses, yeasts, fungi, and parasites) and prions; (ii) antibiotics; (iii) genetically modified cells; (iv) production of secretion products (e.g., signaling molecules with possible interaction with human receptors with hormonal effect), new products (allergens, unknown safety effect). (2) Storage: (i) microbial contamination (bacteria, viruses, yeasts, fungi, parasites), prions; (ii) residues of cryoprotectants. (3) Cell culture: microbial contamination (bacteria, viruses, yeast, fungi, parasites); antibiotics; unintended changes compared to the original cell type; tendency of adult stem cells to transform malignant in long-term culture). (4) Culture medium: microbial contamination (bacteria, viruses, yeasts, fungi, parasites), prions, remaining protein impurities or pyogens (e.g., endotoxins); antibiotics; residue of added exogenous compounds (e.g., growth factors); allergens. (5) Scaffolds, microcarriers and chemical or enzymatic dissociation: scaffolds and chemical cell separation techniques used; chemically synthesized materials, chemical and enzymatic crosslinking agents (e.g., photoinitiators, aldehydes), residues or degradation byproducts thereof. (6) Migration or residues of organic and inorganic substances from disposable products (e.g., plastics, filters), coatings (e.g., equipment), packaging, and cleaning products. (7) New ingredients or compounds present in higher concentrations than in conventional meat production (e.g., protein hydrolysates as potential allergenic compounds); similar hazard to conventional meat production.	(1) (i) Isolation of animal-derived cells from low-risk animals; source animals certified as disease-free; selection of target tissues (e.g., higher chance of prions in tissues such as brain, blood, etc. from affected animals; thorough inspection of source animals and biopsied tissues and cells; (ii) assessment of whether it is permitted and present at safe levels for consumption; (iii) safety assessment of genetically modified cells according to regulatory aspects; (iv) agent/residue testing and safety assessment. (2) (i) Carrying out tests for detection of microorganisms; controlling storage time and temperature; (ii) storing cell banks in vapor media (instead of liquid nitrogen); evaluating possible alternative cryoprotectants already used as food processing aids; carrying out agent/residue testing; safety assessment. (3) Isolation of animal-derived cells from low-risk animals; sterile and controlled conditions, fresh vials of cells stored for each new batch; assessment of possible alterations; agent/residue testing; safety assessment. (4) Isolation of compounds from animal-derived cell media (e.g., FBS) from low-risk animals or non-animal sources; serum substitutes/alternatives or serum-free media; washing/flushing steps; care with recycling/reuse of cell culture media (risk of bioaccumulation of unwanted agents or compounds); sensors; agent/residue testing; safety assessment. (5) Food grade scaffolds and reagents; appropriate washing/discharging steps; agent/residue testing; safety testing. (6) Use of materials/solutions permitted for use in food production; agent/residue testing. (7) Treatments (heat, high pressure, irradiation, etc.) and evaluation of the effects of treatments; monitoring/testing.
FAO and WHO [25]	2023	Italy	Revision	To synthesize the literature on the terminology and production of cultured meat, as well as identify potential hazards related to the cultured meat production process.	(1) Microbiological contamination by fungi, bacteria, viruses, mycoplasma, and prions. (2) (Epi)genetic drift in cell lines, where mutations accumulate over time and can cause changes in phenotypes. (3) Introduction of enzymes or chemicals for the dissociation of microcarriers in the cell harvest (proteases, non-enzymatic dissociation agents, such as dextran sulfate, N-acetyl-L-cysteine and dithiothreitol, or chelating agents, such as EDTA). (4) Toxins and allergenic potential of cell lines, ingredients and additives used in the production of cultured meat. (5) Occurrence of oxidation processes (e.g., lipid) or undesirable biological degradation by enzymatic or thermal action during the processing and storage of cultured meat. (6) Presence of antibiotics. (7) Foreign materials or objects (e.g., plastic, metal, hair, jewelry, glass, etc.) from personnel, equipment, packaging materials present in the final product, resulting in physical harm to the consumer. (8) Microplastic and heavy metals. (9) Physicochemical transformations of food components during interactions with other substances that lead to changes in the compound structure, generating undesirable occurrences of reactive species and other compounds that are harmful to health. These transformations may result from food processing after cell harvesting (e.g., smoking, heat treatment, chemical treatment) or during sterilization of inputs during production (e.g., irradiation) in substances that are sensitive to the method used. To characterize the hazard, the transformed compounds must be detected in the final product and present in the final product at a dangerous level for human consumption.	(1) Perform cell culture under aseptic conditions to reduce or eliminate the use of antibiotics; alternatives to antibiotics to prevent microbial contamination may include the use of approved chemical preservatives, such as sodium benzoate or other antimicrobial compounds; perform early detection of infections in cell cultures through regular monitoring; follow good hygiene practices throughout the production process, such as standard cleaning and sterilization practices for equipment; replace animal-derived components with non-animal-derived components. (2) Use cell banks with quality control of cryopreserved cell lines to mitigate the risk of loss of cell line fidelity due to genetic drift, as well as to protect against the presence of viruses, bacteria, yeasts, and mycoplasma. (3) Quantify and evaluate the hazards associated with the products used. (4) All additives, ingredients, nutrients, and other added substances must be approved for use (e.g., deemed safe and permitted for the specific cell-based food) and must follow potential allergen labeling requirements. Identify any allergens or toxins that may be present in the final product, whether they originate from the cell mass, scaffold material, or processing of the final product. (5) Evaluate the formation of unwanted products resulting from oxidative processes or biological degradation. (6) Quantification of antibiotic residue levels in the final product. (7) Adoption of good practices; inspection of equipment and components; continuous monitoring of cell cultures. (8) Adoption of good practices; filtration, quality control of raw materials; reduction of plastic use; use of food-grade equipment and packaging materials; reduce the use of metals in contact with food; quantification of heavy metal levels in the final product; animal source testing for heavy metals prior to biopsy. (9) Conducting a safety assessment of the final product, including analysis of the chemical transformation of the main food components that must be tested on new ingredients with no history of safe use for use in food. Evaluation of ingredients (in silico and in vitro) to track the reactivity of the components. If it is identified that the transformations pose a risk to human consumption, there is a need for control measures to prevent the occurrence of these dangerous transformations.
Gu, Li, Chan [29]	2023	China and Singapore	Narrative review	Summarize the possible risks that may be introduced during the production of cultured meat and propose countermeasures.	(1) Cell harvesting: (i) Unintentional harmful genetic alteration, and genetic drift can occur at any stage of cell growth, proliferation, differentiation, and subsequent maturation. Genome instability and genetic drift can lead to the production of undesirable substances in the final product at levels that may pose food safety risks; (ii) microbiological contamination (bacteria, fungi, and viruses). (2) Cell culture: (i) use of bovine serum (undefined and variable composition); (ii) cell culture media originating from animals may harbor viruses, prions, and bacteria; (iii) presence of allergens in the culture media. (3) Biomass accumulation: (i) genetic modification and tumorigenicity; (ii) use of non-edible and non-degradable scaffolds. (4) Processing of cultured meat.	(1) (i) To monitor and mitigate genetic drift, cells can be tested using genetic stability assays and chromosome analyses, while freshly stored starting cells can be used for each batch; (ii) verification and certification of the health status of the animal from which the cells will be collected; avoid collection of cells from the brain, spinal cord, lymphoid tissue, tonsils, appendix, enteric nervous system, and blood, as they are more likely to contain prions; adoption of standard operating procedures for sterilization and microbiological examination. (2) (i) Use of bovine serum-free myocardium with chemically defined components; (ii) use of culture media free of viruses, prions, and bacteria; (iii) use of existing materials that have been authorized for food production, which meet the corresponding specifications or criteria, otherwise toxicological tests need to be performed to evaluate the residues of new components in the final product. (3) (i) Monitoring of genetic drift, genetic stability, and potential tumorigenicity, assays should be performed to evaluate the safety of genetically modified cells and their products; (ii) use of edible and non-degradable scaffolds should be separated from the cells before myogenic differentiation or use of edible or degradable scaffolds.
Jones et al. [46]	2023	United States	Experimental study	To evaluate the efficacy of a modified decellularization protocol that eliminates or replaces the unregulated food-grade solution in the production of decellularized plant scaffolds for application in cultured meat production.	(1) Organic solvents (hexanes) and non-ionic detergents (triton X-100-TX100) used in decellularization in scaffold production.	(1) Replace with FDA-regulated solvents for use in food (polysorbate 20-PS20 and sodium dodecyl sulfate). According to the FDA, sodium dodecyl sulfate, PS20, and sodium hypochlorite can be present in a food product at levels below 125, 15, and 200 ppm, respectively. Therefore, it is critical to know the final concentration of all reactants in the process. If the final concentration of each reactant exceeds the specification, additional extraction methods may be required to bring the concentrations within specification.
Ketelings et al. [47]	2023	Netherlands	Qualitative exploratory study on the safety of cultured meat	Explore the barriers to and drivers for the safe introduction of cultured meat into the market.(Semi-structured interviews with stakeholders involved in the production and regulation of cultured meat.)	(1) Unintentional genetic modification in the initial cell line. (2) The medium and serum used for cell proliferation and differentiation, microcarriers, and scaffolds may leave residues. (3) Consumer misinformation about the origin and quality of the product.	(1) Perform genetic stability assays: collect cells and perform chromosomal analyses and other sequencing studies to understand the degree of change that has occurred. (2) Carrying out residual testing based on toxicological standards to attest to product safety. (3) Establish labeling criteria. (4) Establishment and use of a specific monitoring system (HACCP) to ensure the safety of cultured meat.
Ong et al. [48]	2023	United States, Canada, Netherlands	Exploratory study through interviews and workshop discussions with government scientists and regulators from regulatory agencies in several jurisdictions	Identify priorities for research and development of safety-critical methods, datasets, and standards for cultured meat.	(1) Use of inputs unfit for human consumption. (2) Uncontrolled cell proliferation and related concepts of tumorigenicity. (3) Metabolite formation and toxicity of metabolites formed. (4) Genomic and metabolomic instability of cells. (5) Endogenous production of hazardous substances such as toxins or allergens. (6) Possibility that the nutritional profile of the final product (cultured meat) is different from that which it is replacing (conventional meat). (7) Allergenicity. (8) Genetic modifications that may result in metabolites or allergens that are dangerous for human consumption.	(1) Identify exposure routes and establish safe limits for each input used in cultured meat production; inputs that are new require a full risk assessment. (2) Although it is unlikely that a pluripotent or immortalized cell would survive and form tumors after consumption, careful risk communication or testing is needed to resolve any scientific questions about tumorigenicity. (3) Characterize and quantify the stability of inputs and potential metabolites produced during production, storage, or consumption of the product; establish shelf life and recommendations for handling and preparation of the product, limiting the potential for formation of harmful metabolites. (4) Establish methods to detect changes in cell stability when there is a deviation from standard operating protocols; define safe limits for maximum passage; and establish appropriate parameters to monitor and characterize cell stability. (5) Characterize cell lines and identify potential expressions of toxins or allergens. (6) Evaluate the nutritional quality of cultured meat; if necessary, add nutrients to present a nutritional profile similar to that of the reference product; establish identity standards for the developed product. (7) Avoid introduction of known allergens; identify allergens in cultured meat using well-known methods, such as immunoassays; screen new proteins for similarity to known allergens based on molecular weight and stability. (8) Perform screening at genomic, proteomic, or metabolomic levels to identify mutations or changes in cells that may result in dangerous metabolites or allergens.
Sant’Ana et al. [49]	2023	Brazil	Book	Develop and apply a HACCP plan to a cultured meat product (burger).	(1) Medications used in the cell donor animal. (2) Foodborne pathogens from the donor animal (Brucella abortus, Mycobacterium sp., prion–Bovine Spongiform Encephalopathy (BSE), Toxoplasma gondii; Shigatoxigenic *Escherichia coli* (STEC), *Salmonella*, *Cryptosporidium parvum*). (3) Pathogens from muscle sampling, cell transport, preparation of culture media, reception of muscle sampling, cell isolation, cell selection, cell expansion, cell storage, cell thawing, cell inoculum production, microcarrier preparation, gas filtration, cell inoculation and cell biomass production, cell harvesting, and storage and distribution of final product (STEC, *Salmonella*, *L. monocytogenes; Staphylococcus aureus*). (4) Penicillin–Streptomycin (PS) in the preparation of the culture medium. (5) Foreign materials (plastic, metal, insect fragments and stones). (6) Allergens. (7) Fetal Bovine Serum (FBS) (Prions, *L. monocytogenes*, *S. aureus*, *Bacillus cereus*, *STEC*, *Salmonella*, *B. abortus*). (8) Albumin residues can be carried over into the final product and cause allergic reactions when handled or consumed. (9) New allergenic or hazardous substances generated by unintended effects of immortalization. (10) Basic fibroblast growth factor (FGF), insulin-like growth factor-1 (IGF-1), EGF (epidermal growth factor)-residues can potentially reach the final product, becoming a health risk or triggering an allergic reaction when handled or consumed.	(1) Follow the withdrawal period established for each drug. Veterinary inspection (requires veterinary drug residue analysis reports). Follow relevant good practices. (2) Donor animal health report. Animal health inspection before sampling. Follow relevant good practices. (3) Sampling: Perform trichotomy, cleaning, and antisepsis of the semimembranosus muscle region of the animal before collection. Sanitary inspection of the collected tissue for signs of infection. Use sterile material for tissue collection. Transportation: Control the sample storage temperature (4 °C) and transportation time (2 h). Use a sterile culture medium to maintain sample viability during transportation. Add antibiotic at the appropriate concentration. Follow relevant good practices. Preparation of culture media and thawing cells: Perform aseptic handling of cell culture inputs. Follow other relevant good practices. Reception of cell sampling, preparation and isolation of cells: External decontamination of the thermal box and plastic bag. Check sample temperature and preservation conditions. Visual inspection of the material (turbidity, color, and viscosity). Apply validated sterilization procedure. Control the flow rate of the medium and the pressure of the filters during sterilization. Aseptically handle cells and cell culture materials. Follow relevant good practices. Cell sorting: Clean the cell separator. Aseptically handle cells and cell culture materials. Follow relevant good practices. Cell expansion: Regular visual inspection of cultures using a microscope. Examination of cell morphology: signs of deterioration (e.g., granularity, detachment, and vacuolization) and signs of contamination (e.g., medium turbidity, color, and viscosity). Aseptically handle cells and cell culture materials. Follow other relevant good practices. Cell storage: Control storage temperature (≤−80 °C). Label cryovials correctly and check for leaks. Quarantine cells until their origin has been authenticated and they are demonstrated to be free of microorganisms. Aseptically handle cells and cell culture materials. Follow other relevant good practices. Cell inoculum production: Regular visual inspection of cultures using a microscope. Examination of cell morphology: signs of deterioration (e.g., granularity, detachment, and vacuolization) and signs of contamination (e.g., medium turbidity, color, and viscosity). Aseptic handling of cells and cell culture inputs. Follow relevant good practices. Microcarrier preparation: Control storage temperature (4 °C). Aseptically handle microcarriers and inputs. Follow other relevant good practices. Gas filtration: Adoption of a filter maintenance program. Cell inoculation and cell biomass production: Monitor for signs of contamination (e.g., pH, high consumption of alkaline solution for pH maintenance, turbidity, and color). Aseptically handle cells and inputs. Follow other relevant good practices. Cell harvesting: Control temperature and pH of biomass before harvest. Aseptic handling of cells and cell culture inputs. Follow other relevant good practices. Storage and distribution of final product: Temperature control (≤−18 °C) of the product. Follow relevant good practices. (4) Apply a validated washing procedure to remove PS or reduce its concentration. Training in food handling. Follow other relevant good practices. (5) Adoption of a supplier qualification program. Adoption of an equipment maintenance program. Follow other relevant good practices. (6) Food allergen labeling. Adoption of an allergen control program. Adoption of a supplier qualification program. (7) Control storage temperature (≤−10 °C). Adopt a supplier qualification program. Follow other relevant good practices. (8) Food allergen labeling. Apply a validated washing procedure to remove albumin residues or reduce their concentration. Adopt an allergen control program. (9) Regular inspection of cultures by examining cell morphology and signs of culture disruption (e.g., altered growth and viability). (10) Control formulation to ensure minimum levels are used for effective action; apply a validated washing procedure to remove chemical compound residues. Adoption of a residual testing program.
Smith-Uchotski, R.; Wanjiru, P. [50]	2023	United Kingdom	Report	Identify the risks in meat produced by cell culture.	(1) Nutritional: possibility that the nutritional profile of the final product (cultured meat) is different from that which it is replacing (conventional meat). (2) Contamination by components used in cell culture. (3) Infection of cell cultures by bacteria, yeasts, fungi, and viruses introduced unintentionally into the culture system, or through cross-contamination. (4) Risks associated with the cell line (type of cell line, process of differentiation, and maturation of cells), use of cell lines from animals not commonly consumed in the country, increasing the risk of transmission of new diseases and viruses, as well as potential allergenic reactions to new proteins.	(1) Assess the content and quality of nutrients present in cultured meat; if necessary, add nutrients to present a nutritional profile similar to that of the reference product; in the event of a discrepancy in the nutritional composition concerning its counterpart (natural meat), the consumer must be informed to enable him/her to decide whether or not to consume the product. (2) Investigation of how these compounds can bioaccumulate in the final product and how they are removed or whether they are at an acceptable exposure level; to avoid contamination by plastics or metals of the bioreactors, these must be rigorously sterilized and cleaned using, for example, heated steam, sodium hydroxide, and 70% ethanol solution; manage the cleaning of materials so as not to leave residual chemicals; remove toxic products for human consumption used in any of the production stages of cultured meat and assess the risk of exposure to residues of antibiotics and fungicides for human consumption. (3) Adoption of good practices to prevent infection; control of infections with antibiotics and fungicides (assess safety and elimination); in the event of infections, it is necessary to take rapid action to protect neighboring cultures and bioreactors and prevent the infection from spreading; strict quality control and critical control point measures should be adopted to eliminate infected cells, ensuring clean and sterile work surfaces. Corrective actions should also be adopted, such as discarding the infected batch, training operators, sanitizing equipment, and better monitoring. In the case of mycoplasma infection (gram-negative bacteria that do not have a cell wall and do not respond to the effects of many antibiotics), regularly screen cell lines for mycoplasma infections through tests, such as DNA staining, fluorescent staining, and PCR detection; in the case of mycoplasma infection, cells should be disposed of using autoclaving, incineration, or disinfection and disposal. It is possible to decontaminate the infected cell line, but this is a more difficult practice and must ensure adequate cell development and product safety with total disinfection of the mycoplasma. (4) Predicting specific potential hazards that may arise from using different cell lines or cell line-specific risks is complex, as each company will likely be working with its own cell line, which will have its own specific risk considerations. In general, it is necessary to ensure sufficient control over the process to correctly differentiate and mature the cells to produce a final product comparable to traditional meat. Even if the cells may have been differentiated correctly, the maturation of the cells may be incomplete, and they may not have the composition that would be expected for a natural muscle fiber. This may not be a problem depending on the target nutritional profile, but this information needs to be available and there must be no false claims made in the marketing of the product. Another potential hazard is that the cells may not differentiate properly, producing cells of inadequate quality or producing cells of a different cell type (e.g., carcinogenic cells). Therefore, there needs to be some form of quality assurance to ensure that the desired differentiation and maturation is occurring and that the cells that make up the final product are verified (e.g., using biomarkers to track differentiation or genetic techniques). Using cell lines from animals that are not commonly consumed in the country may also pose a risk, increasing the risk of transmission of new diseases and viruses (if the cell lines are not adequately tested and authentic), as well as potential allergenic reactions to new proteins. Proper tracking of the cell line and determination of hazards must be carried out, as well as communication to the consumer about the product being consumed.
Sun et al. [51]	2023	United States	Experimental study	To examine the effects of exposure to microplastics, represented by fluorescent polyethylene microspheres (10–45 µm) on cellular performance, including cell proliferation, cell viability, gene expression, and differentiation processes critical for the production of cultured meat.	(1) Presence of microplastic in cells of marine animal origin. (2) Presence of microplastic in laboratory environments used in the production of cultured meat.	(1) Detection of microplastic in source cells. (2) Detection of microplastic in the final product and assessment of potential long-term impacts on human health.
Zaitseva et al. [52]	2023	Russia	Narrative review	Identify potential consumer health risks and analyze critical control points (CCPs) in cultured meat production.	(1) Phase 1 (cell line development): contamination of the material collected in the biopsy by microorganisms (2) Phase 2 (cell proliferation): (i) addition of nutrients or unintentional contamination by other substances; (ii) addition of potentially allergenic substances for muscle formation; (iii) contamination by pathogenic microorganisms and stem cell death. (3) Phase 3 (cell differentiation of tissue): contamination of the medium by pathogenic microorganisms and death of cellular tissue. (4) Phase 4 (post-processing): (i) change in the biological value of proteins; (ii) contamination of the final product with (physical) residues that may harm the consumer; (iii) contamination by pathogenic microorganisms during removal from the bioreactor; (iv) contamination of the product by pathogenic microorganisms during the packaging process and growth of pathogenic microorganisms in the final product caused by inadequate packaging; (v) migration of chemical contaminants from the packaging to the product; (vi) inclusion or development of compounds that are potentially allergenic to consumers.	(1) Veterinary control of the animal selected for biopsy and control of asepsis during the biopsy. (2) (i) quality control through certification of components added to culture media; (ii) use of hypoallergenic components; control of dissociation of particles added during cell growth; (iii) control of the sterility of the culture medium. (3) Control of sterility of cell culture medium. (4) (i) Evaluation of the composition of amino acids formed in cultured meat and control of the content of essential amino acids in cultured meat; (ii) quality control of the final product; (iii) control of the sterility of the bioreactor and the final product; (iv) sterility control of the product before packaging and of the product packaging, control of pathogenic microorganisms in the packaged product; (v) monitoring of chemical contaminants in the packaged product (batch sampling) and assessment of the safety of the potential contaminant for the health of the consumer; (vi) providing information on the presence of allergens in the packaging of the final product.
Garcia et al. [24]	2022	Brazil	Revision	Develop a regulatory study on alternative proteins in Brazil, specifically on cultured meat.	(1) Cells obtained for culture may carry viruses, bacteria, or prions. (2) Use of contaminated liquid nitrogen in cryopreservation. (3) Use of dimethyl sulfoxide (DMSO) as a cryoprotectant. (4) Molecular and biochemical variations of the cell. (5) Use of hormones in the culture medium. (6) Use of antibiotics in the culture medium. (7) Contamination of the culture medium. (8) Material for the production of scaffolding. (9) Contamination of bioreactors. (10) Genetic modification.	(1) Obtaining cells from healthy animals; keeping track of the animal’s breeding history (sex, age, biopsy site, medical history, collection day, and protocol used); performing pre-biopsy inspection by a veterinarian; the cells obtained by biopsy must undergo checking procedures and their DNA evaluated by techniques, such as PCR, to detect the presence or absence of endogenous retrovirus; the manufacturer must take all precautions for handling these cells, not only concerning their quality and asepsis, but in protecting the handler. (2) Checking the sterility of liquid nitrogen or cells intended for storage to prevent unintentional spread in future batches. (3) Verification of the amount of DMSO in the final product for safety assessment; replacement of DMSO with inulin or sorbitol. (4) Molecular and biochemical variations of the cultured cell can be minimized by controlling the number of passages and renewing the cell *pool* from a cell bank, after genetic variations have been recorded; conducting research using sequencing techniques for genetic comparison between the original and final cells, helping to identify potentially dangerous compounds. (5) Identification and determination of hormone concentration in the final product, meeting the same requirements established for conventional meat. (6) If used, the antibiotic must be applied in low concentrations, only in the initial stages of the meat manufacturing process, accompanied by periodic rinsing so that it does not persist in the final product; if present in the final product, the antibiotic residue must meet the same requirements established for conventional meat. (7) Continuous monitoring for contamination by microbiological agents, to ensure sterility throughout the meat production process in bioreactors; parameters, such as pH control or dissolved oxygen level, can be used to assess signs of contamination; cleaning and sterilization of bioreactors and equipment with products that do not contain any toxic residue that could compromise the quality of the meat produced. (8) It must be edible, biodegradable, palatable, and safe during the cultivation process and after cooking and preparation of the products; if it is not edible or biodegradable, it requires mechanical, enzymatic, or chemical removal of the scaffolds; if the materials are not yet approved for use in food, they will require an individual safety assessment, as required for any food additive or ingredient. (9) Adequate asepsis of bioreactors; use of automated and closed systems. (10) Assess the risk of transferring these modified genes to the consumer (e.g., antibiotic resistance genes).
Fernandes et al. [53]	2021	Brazil	Narrative review	Map the technological development of cultured meat from the perspective of cellular agriculture, using patent family records, start-ups, and their representative investors as indicators of R&D patents.	(1) Cell line development: genetic approaches to induce pluripotency. (2) Cell culture medium: animal components (serum) and antibiotics. (3) Scaffolding: materials that are not biocompatible. (4) Contamination associated with human handling.	(1) Cell line development: development of small molecule cocktails that can replace the need for genetic approaches to induce pluripotency and facilitate maintenance of pluripotency. (2) Development of culture media free of animal components, without antibiotics. (3) Use of biocompatible scaffolding materials (edible and/or biodegradable and food grade). (4) Use of integrated and closed automation systems.
Hadi and Brightwell [54]	2021	New Zealand	Narrative review	Evaluate technological, environmental and regulatory aspects of cultured meat, plant-based meat, insect protein, and single-cell protein.	(1) Bovine serum used in the culture medium with the presence of bovine viral diarrhea virus, reovirus 3, rabies virus, bluetongue virus, bovine adenovirus, bovine parvovirus, and bovine respiratory syncytial virus; bovine serum with the presence of pathogenic and infectious prions. (2) Introduction of different genes.	(1) Use serum from healthy animals; perform tests on the animals; remove contaminants. (2) Use of small molecules as an alternative system for cellular reprogramming.
Soice and Johnston [55]	2021	United States	Narrative review	Define existing methods for producing new cell lines and their limitations.	(1) Oncogenic compounds. (2) Cell line contamination. (3) Cellular mutations that cause phenotype changes. (4) Liquid nitrogen contamination during cryopreservation.	(1) Conduct tests to confirm the absence of tumorigenicity during the cultured meat manufacturing process by sampling a small portion of the cells, which should be an accurate representation of the entire population in a cell line. (2) If a cell line is confirmed as safe for consumption, it will still need to be monitored regularly for contamination and genetic drift. (3) To mitigate the risk of loss of cell lineage fidelity to genetic drift, cell banks should be constructed in which cryopreserved cultures of relevant cell lines are quality controlled and protected against viruses, bacteria, yeasts, and mycoplasma. To create a cell bank, cells must be selected, validated, and cryopreserved in small batches that can be thawed, validated again, and expanded for culture. (4) Storage in the vapor phase rather than the liquid phase may reduce the potential for cross-contamination, because liquid nitrogen has the potential to transfer pathogens to cells even if stored in freezer bags.
Melzener et al. [56]	2020	Netherlands	Narrative review	To evaluate potential scenarios for stem cell collection steps from donor animals by tissue biopsy for cultured meat production.	(1) Contamination of material collected in the biopsy by microorganisms. (2) Genomic alterations that may have negative implications for food safety.	(1) Biopsies should only be performed on healthy animals after inspection by a veterinarian. It is recommended to include a report on the animal’s health status, any veterinary medications administered to the animal in question, specifying the dates of administration and the occurrence of diseases that may affect the safety of the meat; performing a blood analysis of the donor animal and laboratory analysis of the sample itself. (2) Specific tests, such as DNA genotyping, can be employed to ensure that cultured meat products comply with strictly defined safety limits.
Zhang et al. [57]	2020	China	Narrative review	Discuss the advantages and development of cultured meat, technical challenges, and potential strategies to address issues in cultured meat production.	(1) Genetic and phenotypic instability of the cell lineage (2) Contamination by microorganisms	-
Bhat et al. [26]	2019	New Zealand and India	Narrative review	Highlight emerging biotechnology options for thedevelopment of cultured meat and suggest ways to integrate these emerging technologies into meat research.	(1) Antibiotics, antifungals, growth factors, and hormones in culture media. (2) Polyglycolic acid, polylactic acid, and polyurethanes. (3) Use of phenol in cell culture to track pH change.	(1) Assess safety for use in food. (2) Assess safety for use in food. (3) Cannot be used in meat grown for human consumption.
Leśkiewicz, K. [58]	2019	Poland	Narrative review	Establish how to legally qualify in vitro meat from animal cell cultures, as well as whether current regulations ensure that these products are safe for human health.	(1) Lack of sufficient information on labels.	(1) Present information about the origin and components of cultured meat.
Stephens et al. [19]	2017	United Kingdom	Narrative review	Provide an overview of the state of the art of cultured meat technology, discuss the potential benefits of cultured meat, detail the technical challenges faced, and identify key consumer, policy, and regulatory aspects of cultured meat.	-	(1) Identification of the main possible pathogens and safety measures to inhibit contamination (through a HACCP-based system). (2) Monitoring and quality assurance of cellular functions at each stage (viability, self-renewal, death, and differentiation) are essential for quality, function and sustainability, assays for cellular potency, and genetic stability tests. (3) Metabolic waste management by disposal, recycling, or upgrading. (4) Risk and operability study of the cultured meat production plant.
Bhat et al. [59]	2015	India	Narrative review	Address the challenges and benefits of lab-grown meat compared to conventional meat production.	(1) Nutrient disparity compared to conventional meat. (2) Physical and chemical hazards.	(1) Nutrient inclusion. (2) Pay attention to the safety of added substrates and other compounds in the culture medium.
Gunnarsdottir [60]	2015	United Kingdom	Case study	Summarize findings and policies related to cultured meat.	(1) Chemical, physical and biological hazards. (2) Lack of information to the consumer.	(1) Supervision of production and adoption of hygiene standards, nutritional standards, donor categories, cell donor welfare, etc. (2) Consumer protection (product categorization, labeling, product safety, warnings/endorsements, etc.).
Petetin, L. [61]	2014	United Kingdom	Narrative review	Critically analyze the development of a type of cultured meat and assess the risks posed by cultured meat.	(1) Contamination and errors during production. (2) Use of additives, antibiotics and dyes.	(1) and (2) Assessment of risk and regulation for the safe production of cultured meat.
Schneider [62]	2013	United States	Narrative review	Describe regulatory techniques and approaches to cultured meat.	(1), (2), (3), (4) Contamination and adulteration of cultured meat. (5) Use of human cells to produce cultured meat (“victimless cannibalism”).	(1) Presence of regulations similar to those for slaughterhouses to ensure hygienic and sanitary conditions. (2) Imposition of uniform labeling for cultured meats, informing the nature of the product and the additives used. (3) Regulation to ensure that the cultured meat produced is safe for human consumption. (4) Development and implementation of standard operating procedures (SOP) for sanitation and maintenance of suitable and safe environments for cultured meat production. (5) Prohibition and monitoring of the use of human cells to produce cultured meat for human consumption.

**Table 2:** Basic components of culture media for cell cultivation for cultured meat production.

Component	Function
Glucose	Main source of energy, reducing agent of oxidative stress. Glucose demand differs depending on the cell type.
Amino acids	Production of proteins, nucleotides, and short-chain peptides. Cells grown in vitro obtain essential amino acids (arginine, cysteine, glutamine, histidine, isoleucine, leucine, methionine, phenylalanine, threonine, tryptophan, tyrosine, and valine) from the culture medium. Cells capable of synthesizing certain amino acids in vivo do not exist in vitro. Therefore, there is a difference between what is considered “essential” in cell culture and what is considered “essential” in the whole organism. When culturing cells in vitro, the 13 amino acids mentioned above are considered essential, although most of these amino acids are considered nonessential when in vivo.
L-glutamine (specific amino acid)	Major amino acid, contributing to protein biomass and transport into cells. Acts as an alternative source of supplemental energy for cell growth because it can be easily metabolized as a supplemental alternative energy source at the time of growth and proliferation because it becomes a precursor of carbon and nitrogen-containing biomolecules, as intermediate molecules used in the synthesis of various amino acids and nucleotides.
Inorganic salts	Maintenance of cellular osmotic pressure and acting as enzymatic cofactors, receptors, and extracellular matrix proteins (e.g., calcium chloride, potassium chloride, magnesium sulfate, sodium chloride, sodium phosphate, and sodium bicarbonate)
Vitamins	Responsible for cell maintenance and growth. Most vitamins are usually supplied through cultures as a single compound that cells can absorb directly. Some can act as enzyme cofactors, antioxidants, or hormones.
Buffer solution	Maintains the pH of the cell culture (e.g., bicarbonate system or hydroxyethyl piperazine ethane sulfonic acid-HEPES)
Serum	The primary cell growth factor in mammals. Serums contain attachment factors, macronutrients, micronutrients, growth factors, hormones, and protective elements, which promote rapid cell growth, but also carry the risk of contamination by viruses or prions (infectious proteins that can cause serious and fatal diseases in humans and animals).
Serum-free media	They are generally composed of basic cultures and supplements to which chemical components or growth factors may be added. In general, supplementary components can be divided into essential factors (transferrin and insulin) and special factors (adhesion factors, binding proteins, and hormones). Compared to serum-containing media, serum-free media still have lower performance in terms of growth promotion and differentiation efficiency; however, studies have advanced towards the production of more efficient serum-free media.
Hormones and growth factors	Stimulation of cell growth, proliferation, and differentiation (e.g., insulin, cortisol, growth hormone, parathyroid hormone, triiodothyronine (T3), thyroxine (T4), thyroid hormone, follicle-stimulating hormone, testosterone, progesterone, prolactin, and lutein).
Recombinant proteins	Contributes to increasing production efficiency in the development of cultured meat

**Table 3:** Characteristics of the different types of strategies for tissue structuring.

Type of Fabric Structuring	Features
Microcarriers	Composed primarily of polystyrene, cross-linked dextran, cellulose, gelatin, or polygalacturonic acid (PGA) and coated with collagen, peptides. They are simple to produce and allow for the efficient increase of adherent cells (large-scale cell proliferation). Use for suspension culture. Can be used in floating incubators. They can be used as a temporary carrier to support cell proliferation, with subsequent removal and processing of the cells; as a temporary carrier that is dissolved or degraded to release the cells; or as an edible carrier incorporated into the final product. Use of non-edible microcarriers will require a dissociation step, which will likely increase costs and may introduce safety concerns.
Porous scaffolding	Easy production and low-cost. They have a sponge-like structure that provides the mechanical stability necessary for the seeded cells to form tissues and deposit extracellular matrix. They present empty spaces through which the culture medium can circulate. Provide a robust framework for cell growth. The distribution of pore sizes can be adjusted to influence cell differentiation in the desired direction and facilitate the transport of oxygen, nutrients, and waste products. Used in the maturation and differentiation phase of tissues.
Fiber scaffolding	They present empty spaces through which the culture medium can circulate. They have the ability to support both cellular adhesion to the fibers and the diffusion of oxygen and nutrients through the spaces between the fibers. They are best suited to induce muscle fiber alignment. They have long, thin fibers (usually produced by electrospinning or rotary jet spinning). Spinning techniques can be applied to various biomaterials. They have the ability to produce aligned fibers that can help promote muscle fiber maturation and tissue differentiation.
Hydrogels	It is a hydrophilic polymer matrix with a high water retention capacity. They mimic the structure of the naturally occurring extracellular matrix, increasing cellular compatibility. The polymeric matrix must be cytocompatible, made of biomaterials that are not toxic to cells. Requires little scaffolding material, reducing costs.
3D Bioprinting	It can be used to produce defined structures at different scales. Precise control over cell and bio-ink positioning. The bioink must be edible or capable of completely biodegrading during the culture period into edible components. The main categories of 3D bioprinters are extrusion, laser, and inkjet.
Scaffold-free production	They are based on sheets of cells or organoids. In the case of cell sheets, it requires labor for stacking multiple sheets. Do not introduce any exogenous scaffolding material. They depend on cells to secrete their own extracellular matrix. It may be advantageous from a regulatory point of view as it presents fewer hazards. It has greater organoleptic and nutritional similarity with conventional meat.

**Table 4:** Hazards in cultured meat production and process control actions to avoid or reduce exposure to the hazard.

Phase	Potential Hazard	Control Measures to Avoid or Reduce Exposure to Hazard
Collection and development of cell line	Health status of the animal whose cells will be collected	Cell collection from disease-free animals. To meet these requirements, animals eligible for biopsy must be in a veterinarian-certified healthy condition, similar to the procedure for animals slaughtered for traditional meat production. Assess the health status of the animal; document any veterinary medications administered to the animal, such as antibiotics (including dates of administration and the established withdrawal period for each medication); and identify any diseases that could potentially affect the safety of the resulting cultured meat product. Application of advanced laboratory techniques for in-depth analysis of biopsy samples and harvested stem cells to ensure a disease-free baseline state in cultured meat production. The supplier confirmation that the cell line has tested negative for known infectious agents affecting the donor animal. Obtaining cells from animals free from potential toxins (mercury, ciguatera toxin and harmful algae toxins if the cells are from marine organisms). Adhere to good cell culture practices (GPCC).
	Donor tissues and/or blood may contain pathogens such as prions	Avoid using cells from tissues, such as brain, spinal cord, lymphoid tissue, tonsils, appendix, enteric nervous system, and blood from infected animals.
	Exposure to contaminants in the collection of animal muscle tissue cells	Perform trichotomy, cleaning, and antisepsis of the region of the animal where the biopsy will be performed before collection, as well as subsequent inspection of the sampled tissue for signs of contamination.
	Contamination of stem cells by chemicals or recombinant proteins	Assess the safety and legality of using chemicals or recombinant proteins in food.
	Contamination of cells by microorganisms	Any vials of propagated cells that are contaminated with microorganisms must be destroyed and the destruction documented. If cells are received from a supplier, request a health certificate and species authentication (DNA barcode).
	Enzymes/chemicals used for cell release and preservation, such as cryoprotectants as dimethyl sulfoxide (DMSO), sorbitol, inulin	Culture medium components need to be evaluated from a food safety perspective.
	Butanediol cryoprotectant	Used in the minimum quantity necessary to achieve the intended effect and quantified in the final product when used.
	Cryoprotectant dimethyl sulfoxide (DMSO)	Assess whether the quantity is less than 2 ppm in the final product.
	Cryoprotectants formamide and methanol	Not allowed in the final product.
	Cryoprotectant residues	Cell bank storage in vapor media as an alternative to liquid nitrogen. Evaluation of possible alternative cryoprotectants already used safely as food processing aids. Performing agent/residue testing. Security assessment.
	Time and temperature of transport and storage of the cell line	Materials subject to cold chain management must be shipped with adequate refrigeration to maintain temperature throughout the shipping cycle, with a safety margin for at least 24 h of delay. For short-term cell transport, a recommended approach involves transferring samples to sterile tubes containing Dulbecco’s Modified Eagle Medium (DMEM) with 1% penicillin–streptomycin (PS). These tubes can then be placed in a cooler at 4 °C with a suitable cooling agent, such as ice. Dry ice can also be used. For long-term transport, cryopreservation is the leading approved method. Transport is accomplished using dry carriers containing absorbent material, which can be filled with liquid nitrogen to sustain vapor phase temperatures for up to 2 weeks if properly loaded.
	Use of animal cell culture components and carriers that have no history of safe use in the cultured meat production process	Evaluate the safety of the products used. Factors such as container strength, pressure resistance, temperature controls, choice of refrigerants (whether liquid nitrogen or dry ice), and the heat transfer capability of the container material must be continually evaluated and tested. Perform a new test upon arrival of the cells at the new destination to detect any potential contamination that may have occurred during the transport process. Establish a protocol for cell bank stability, including a table to record test results. Cell bank reports should be fully documented and updated as necessary.
	Antibiotics	Assess whether it is permitted and whether it is present at safe levels for consumption. It should be applied in low concentrations. Use only in the initial stages of the meat manufacturing process, accompanied by periodic rinsing, reducing the chances of them persisting in the final product. The residual antibiotics in the final product must meet the requirements established for conventional meat.
	Genetically modified cells	Cells can be tested using genetic stability assays and chromosome analyses to monitor and mitigate genetic drift, while freshly stored starter cells can be used for each batch. Monitoring genetic drift, genetic stability, and potential tumorigenicity. Safety assessment of genetically modified cells and their products according to regulatory aspects.
	Genomic and metabolomic instability of cells	Characterize cell lines and identify potential expressions of toxins or allergens.
	Production of secretory products (e.g., signaling molecules with possible interaction with human receptors with hormonal effect), new products (allergens, unknown safety effect)	Agent/residue testing and safety assessment.
	Heavy metals	Animal source testing for heavy metals before biopsy.
Proliferation	Addition of nutrients or other compounds used in cell proliferation (amino acids, vitamins, minerals, growth factors, antibiotics, etc.)	Culture medium components need to be evaluated from a food safety perspective. Quality control through certification of components added to culture media.
	Microorganism contamination	Control of sterility of cell culture medium. To prevent the spread of microbial contamination, it is essential to protect nearby bioreactors. Adoption of good practices to avoid infection. Infection control with antibiotics and fungicides (assess safety and elimination). Even if the microorganism is identified and its susceptibility to antimicrobials is known, the use of chemicals may not be the ideal solution. Discarding the batch to prevent potential risks to consumers is advisable. Rapid action must be taken in the event of infections to protect neighboring cultures and bioreactors and prevent the infection from spreading. Strict quality control and critical control point measures must be adopted to eliminate infected cells, ensuring clean and sterile work surfaces. Corrective actions: disposal of the infected batch, operator training, equipment sanitation, and better monitoring. In the case of mycoplasma infection (gram-negative bacteria that lack a cell wall and do not respond to the effects of many antibiotics), regularly screen cell lines for mycoplasma infections using tests, such as DNA staining, fluorescent staining, and PCR detection. In the case of mycoplasma infection, cells should be disposed of using autoclaving, incineration, or disinfection and disposal.
	Recycling/reuse of cell culture media (risk of bioaccumulation of unwanted agents or compounds)	In case of reuse, agent/residue testing and safety assessment are required.
	Growth factors, hormones, microorganisms, and endotoxins present in fetal bovine serum (FBS) used in culture media	Use of proven safe FBS for cultured meat production. Use of FBS-free media with chemically defined components. Carcinogenicity and toxicity testing when these materials are used. Use of myo without bovine serum; use of culture media free of viruses, prions, and bacteria.
	High ammonia production in the cell proliferation and differentiation phases (in cultured fish meat)	Use of glutamine substitutes, including α-ketoglutarate (αKG), glutamate (Glt), and pyruvate (Pyr) had a positive impact on cell proliferation and differentiation by reducing the rate of ammonia production. Collect reactor samples to measure ammonia, nitrite, and nitrate as part of GMPs.
	Presence of allergens, such as parvalbumins, tropomyosin, arginine kinase, and myosin light chains (in cultured fish meat)	Selective cultivation of specific cell types (e.g., myogenic and adipogenic cells) to avoid allergenic components. Genetic modifications, such as the use of RNA interference (RNAi) techniques to eliminate causative genes. Incorporation of additives such as creatine or ethylenediaminetetraacetic acid (EDTA) into the cell culture medium modulating the expression of allergens (e.g., parvalbumin), to reduce allergenicity.
	Contamination by potentially allergenic compounds	Use existing materials authorized for food production, and which meet the corresponding specifications or criteria. Otherwise, toxicological tests must be carried out to assess the residues and allergenic potential of new components in the final product. Use of hypoallergenic components. Control of dissociation of added particles during cell growth. Potentially allergenic compounds should be stored in a separate area.
	Bioreactor: Rapidly growing bacteria, yeasts, and fungi can contaminate meat culture bioreactors through contaminated components, air filtration failures, poor cleaning, and incomplete sterilization of equipment, piping, and fittings, or during handling operations when strict aseptic techniques are not followed	Daily testing for mycoplasmas and microbial testing for other pathogens every two weeks. To avoid contamination by plastics or metals of the bioreactors, they must be rigorously sterilized and cleaned using, for example, heated steam, sodium hydroxide, and 70% ethanol solution. Manage the cleaning of materials to avoid leaving residual chemicals. Remove toxic products for human consumption used in any of the production stages of cultured meat and assess the risk of exposure to antibiotic and fungicide residues for human consumption. After removing chemicals from a bioreactor, perform UV sanitation and collect samples for sanitation verification.
	Genetic modifications to alter the expression levels of certain nutrients	Monitoring genetic drift, genetic stability, and potential tumorigenicity. Safety assessment of genetically modified cells and their products according to regulatory aspects.
	Contamination by foreign materials (physical)	Removal of material, if present. Regularly checking the biofilter to remove any solid materials.
	Intentional contamination	Safety and monitoring; safety program and policy; staff training; only authorized personnel have access to the production room and process control room.
	Allergenic potential of components used as a support structure (scaffold) for cell growth, proliferation, and differentiation	Use of hypoallergenic cellular scaffolds.
	Use of non-edible and non-degradable scaffolds.	Edible and non-degradable scaffolds must be separated from cells before myogenic differentiation or use edible or degradable scaffolds.
Cell differentiation	Use of growth factors like TGF, FGF, and IGF for cell differentiation and oxygen carriers like hemoglobin, myoglobin perflurochemicals. Use of enzymes/chemicals (trypsin, EDTA) during cell collection	Growth factors and other components must be evaluated from a food safety perspective.
	Microorganism contamination	Control of sterility of cell culture medium. To prevent the spread of microbial contamination, it is essential to protect nearby bioreactors. Adoption of good practices to avoid infection. Infection control with antibiotics and fungicides (assess safety and elimination). Even if the microorganism is identified and its susceptibility to antimicrobials is known, the use of chemicals may not be the ideal solution. Discarding the batch to prevent potential risks to consumers is advisable. Rapid action must be taken in the event of infections to protect neighboring cultures and bioreactors and prevent the infection from spreading. Strict quality control and critical control point measures must be adopted to eliminate infected cells, ensuring clean and sterile work surfaces. Corrective actions: disposal of the infected batch, operator training, equipment sanitation, and better monitoring. In the case of mycoplasma infection (gram-negative bacteria that lack a cell wall and do not respond to the effects of many antibiotics), regularly screen cell lines for mycoplasma infections using tests, such as DNA staining, fluorescent staining, and PCR detection. In the case of mycoplasma infection, cells should be disposed of using autoclaving, incineration, or disinfection and disposal.
	Different types of materials are used to produce edible and non-edible scaffolding.	The edible scaffolds used cannot be harmful or cause allergies to consumers. Non-edible scaffolding must be biodegradable.
	High ammonia production in the cell proliferation and differentiation phases	Use of glutamine substitutes, including α-ketoglutarate (αKG), glutamate (Glt), and pyruvate (Pyr), had a positive impact on cell proliferation and differentiation by reducing the rate of ammonia production. Collect reactor samples to measure ammonia, nitrite and nitrate as part of GMPs.
	Bioreactor: Rapidly growing bacteria, yeasts, and fungi can contaminate meat culture bioreactors through contaminated components, air filtration failures, poor cleaning, and incomplete sterilization of equipment, piping, and fittings, or during handling operations when strict aseptic techniques are not followed	Daily testing for mycoplasmas and microbial testing for other pathogens every two weeks. To avoid contamination by plastics or metals of the bioreactors, they must be rigorously sterilized and cleaned using, for example, heated steam, sodium hydroxide, and 70% ethanol solution. Manage the cleaning of materials to avoid leaving residual chemicals. Remove toxic products for human consumption used in any of the production stages of cultured meat and assess the risk of exposure to antibiotic and fungicide residues for human consumption. After removing chemicals from a bioreactor, perform UV sanitation and collect samples for sanitation verification.
	Contamination by foreign materials (physical)	Removal of material, if present. Regularly checking the biofilter to remove any solid materials.
	Intentional contamination	Safety and monitoring; safety program and policy; staff training; only authorized personnel have access to the production and process control rooms.
	Allergenic potential of components used as a support structure (scaffold) for cell growth, proliferation, and differentiation	Use of hypoallergenic cellular scaffolds.
	Genetic modifications	Monitoring genetic drift, genetic stability, and potential tumorigenicity. Safety assessment of genetically modified cells and their products according to regulatory aspects. Perform screening at the genomic, proteomic, or metabolomic levels to identify mutations or changes in cells that may result in dangerous metabolites or allergens.
	Use of non-edible and non-degradable scaffolds.	Edible and non-degradable scaffolds must be separated from cells before myogenic differentiation or use edible or degradable scaffolds.
	Organic solvents (hexanes) and non-ionic detergents (triton X-100-TX100) used in scaffold decellularization	Replace with solvents regulated for use in food (e.g., Polysorbate 20-PS20 and sodium dodecyl sulfate). Sodium dodecyl sulfate, PS20, and sodium hypochlorite may be present in a food product at levels below 125, 15, and 200 ppm, respectively. Therefore, it is essential to know the final concentration of all reagents in the process. If the final concentration of each reagent exceeds the specification, additional extraction methods are required to reduce the concentrations to within the tolerated limit.
Final product formation is post-processing	Product contamination due to unhygienic handling practices	Establish standard operating procedures to prevent contamination during the handling and production of cultured meat. Microbial testing of hydrogels and bio-inks. Temperature control of the cell diffusion process.
	Presence of substances used in the extracellular matrix, basic culture fluid, and serum not yet authorized for use in food	Assess whether these materials should be replaced with edible materials or considered waste substances. Safety verification of existing substances.
	Metabolite formation and toxicity of metabolites formed	Characterize and quantify the stability of inputs and potential metabolites produced during production, storage, or consumption of the product; establish shelf life and recommendations for handling and preparation of the product, limiting the potential for formation of harmful metabolites.
	pH of cultured meat (bacterial growth and putrefaction of cultured meat)	Control of the pH of the final product (below 6.0).
	Use of unsuitable packaging materials (waste migration) or incorrect storage of packaging materials	Inspection of packaging materials for signs of inappropriate odor, damage, or contamination. Certification by the analysis provider indicating that the materials are safe for food contact. Storage of packaging materials covered and in a segregated area, away from the rest of the plant, and any allergenic materials or chemicals; temperature control of stored materials. Monitoring of chemical contaminants in the packaged product (batch sampling) and assessment of the safety of the potential contaminant for consumer health.
	Cryoprotectant dimethyl sulfoxide (DMSO)	Assess whether the quantity is less than 2 ppm in the final product.
	Cryoprotectants formamide and methanol	Not allowed in the final product.
	Butanediol cryoprotectant	Used in the minimum quantity necessary to achieve the intended effect and quantified in the final product when used.
	Potentially allergenic compounds	Detailed description of each step of the cultured meat production process based on a flow diagram. Allergen control by label declaration. Provide information on the presence of allergens on the packaging of the final product.
	Intentional contamination	Safety and monitoring; safety program and policy; staff training; only authorized personnel have access to the production and process control rooms.
	Growth of spore-forming bacteria (post-processing)	Proper labeling and thawing instructions; use suitable packaging materials with oxygen transmission rate-10,000 cc/m ^2^/24 h at 24 °C or higher. Process control for temperature of products and transport vehicles with data logger.
	Presence of parasites in cultured fish meat	Freeze and store at room temperature of −20 °C or below for 7 days.
	Consumer misinformation about the origin and quality of the product	Establish labeling criteria.
	Consumption of genetically modified products	Although it is unlikely that a pluripotent or immortalized cell would survive and form tumors after consumption, careful risk communication or testing is needed to resolve any scientific questions about tumorigenicity.
	Possibility that the nutritional profile of the final product (cultured meat) is different from that which it is replacing (conventional meat)	Assess the nutritional quality of cultured meat; if necessary, add nutrients to present a nutritional profile similar to that of the reference product. The consumer must be informed if there is a discrepancy in the nutritional composition compared to its counterpart (natural meat). Establish identity standards for the developed product.
	Hormones	Identification and determination of the concentration of these molecules, meeting the same requirements established for conventional meat.
	Presence of microplastic	Detection of microplastic in source cells. Detection of microplastic in the final product and assessment of potential long-term impacts on human health.
	Presence of heavy metals	Quantification of heavy metal levels in the final product. Animal source testing for heavy metals before the biopsy.
	Change in the biological value of proteins	Evaluation of the composition of amino acids formed in cultured meat and control of the content of essential amino acids in cultured meat.
	Oncogenic compounds	Perform tests to confirm the absence of tumorigenicity of cultured meat through sampling.

**Table 5:** Materials commonly used in scaffolding, their characteristics, potential hazards, and actions to control the hazard.

Materials	Features	Potential Hazards	Actions
Synthetic polymers	They are synthetic materials that do not occur in nature, but have distinct characteristics advantageous for tissue engineering, such as tunability, reproducibility, and being chemically defined. They have adjustable properties and can be used in multiple scaffolding configurations. Little variability between batches. Low cost.	Many synthetic polymers are not edible or biodegradable, while others have slow degradability or unknown degradation profiles.	Use as microcarriers or other temporary scaffolding. Evaluate the final product for safety for consumption.
Self-assembling peptides	They are capable of imitating the native architecture of the extracellular matrix. They can be used for conventional scaffold production methods, as part of bioinks for additive manufacturing or as coatings to aid large-scale cell proliferation. High cost.	Still needs safety assessment for human consumption.	Safety assessment for application in cultured meat.
Extracellular matrix molecule	They provide an environment similar to natural tissues, facilitating cell attachment. They can be produced by animal-derived extracellular matrix, using microbial fermentation, plant molecular farming, or cell-free systems. Low cost.	-	Safety assessment for application in cultured meat.
Materials derived from plants, crustaceans, insects and fungi	Cellulose obtained from plants or produced by fungi. Alginates (polysaccharides derived from algae). Fungal mycelium. Chitosan (polymer derived from insects and crustaceans).	-	Decellularization methods will need to be developed for food safety. Assessment of safety in the production of food for human consumption.

## Data Availability

No new data were created or analyzed in this study. Data sharing is not applicable to this article.

## Supplementary Materials

The following supporting information can be downloaded at: https://www.mdpi.com/article/10.3390/foods14010129/s1, Table S1: Search strategy.
