# Supplementary material for: Global Insights into Cultured Meat: Uncovering Production Processes, Potential Hazards, Regulatory Frameworks, and Key Challenges—A Scoping Review
Source: Foods. 2025 Jan 4;14(1):129. doi: 10.3390/foods14010129 (PMC11720233; doi:10.3390/foods14010129)
Supplement: Supplementary file 1 [file foods-14-00129-s001.zip › foods-3379516-supplementary.pdf]

Table S1. Serach strategy (Medline/Pubmed, 745; Embase, 133; Cochrane Library, 0; BVS, 14; Web of Science, 100; Scopus, 93; Google scholar, 100)

Databases	Search strategy	Results
MEDLINE/Pubmed	(("In Vitro Meat"[MeSH Terms] OR ("In Vitro Meat"[MeSH Terms] OR ("vitro"[All Fields] AND "meat"[All Fields]) OR "In Vitro Meat"[All Fields] OR ("vitro"[All Fields] AND "meats"[All Fields])) OR "meat in vitro"[All Fields] OR ("In Vitro Meat"[MeSH Terms] OR ("vitro"[All Fields] AND "meat"[All Fields]) OR "In Vitro Meat"[All Fields] OR ("meats"[All Fields] AND "vitro"[All Fields])) OR "cell based meat"[All Fields] OR "cell based meat"[All Fields] OR "Cell-Based Meats"[All Fields] OR (("meat"[MeSH Terms] OR "meat"[All Fields]) AND "cell based"[All Fields]) OR (("meat"[MeSH Terms] OR "meat"[All Fields] OR "meats"[All Fields] OR "meat s"[All Fields]) AND "cell based"[All Fields]) OR "Cultivated Meat"[All Fields] OR "Cultivated Meats"[All Fields] OR "meat cultivated"[All Fields] OR ("In Vitro Meat"[MeSH Terms] OR ("vitro"[All Fields] AND "meat"[All Fields]) OR "In Vitro Meat"[All Fields] OR ("meats"[All Fields] AND "cultivated"[All Fields])) OR "Cultured Meat"[All Fields] OR "Cultured Meats"[All Fields] OR "meat cultured"[All Fields] OR ("In Vitro Meat"[MeSH Terms] OR ("vitro"[All Fields] AND "meat"[All Fields]) OR "In Vitro Meat"[All Fields] OR ("meats"[All Fields] AND "cultured"[All Fields])) OR "lab grown meat"[All Fields] OR "lab grown meat"[All Fields] OR ("In Vitro Meat"[MeSH Terms] OR ("vitro"[All Fields] AND "meat"[All Fields]) OR "In Vitro Meat"[All Fields] OR ("lab"[All Fields] AND "grown"[All Fields] AND "meats"[All Fields])) OR "meat lab grown"[All Fields] OR (("meat"[MeSH Terms] OR "meat"[All Fields] OR "meats"[All Fields] OR "meat s"[All Fields]) AND "lab grown"[All Fields]) OR "laboratory grown meat"[All Fields] OR "laboratory grown meat"[All Fields] OR ("In Vitro Meat"[MeSH Terms] OR ("vitro"[All Fields] AND "meat"[All Fields]) OR "In Vitro Meat"[All Fields] OR ("laboratory"[All Fields] AND "grown"[All Fields] AND "meats"[All Fields])) OR (("meat"[MeSH Terms] OR "meat"[All Fields]) AND "laboratory grown"[All Fields]) OR (("meat"[MeSH Terms] OR "meat"[All Fields] OR "meats"[All Fields] OR "meat s"[All Fields]) AND "laboratory grown"[All Fields]) OR ("Animal-free"[All Fields] AND ("meat"[MeSH Terms] OR "meat"[All Fields])) OR (("artificial"[All Fields] OR "artificially"[All Fields]) AND ("flesh"[All Fields] OR "fleshed"[All Fields] OR "fleshes"[All Fields]))) AND ("Risk"[MeSH Terms] OR "Risks"[All Fields] OR "Hazard Analysis and Critical Control Points"[MeSH Terms] OR "Food Safety Analysis"[All Fields] OR "analyses food safety"[All Fields] OR "analysis food safety"[All Fields] OR "Food Safety Analyses"[All Fields] OR ("Hazard Analysis and Critical Control Points"[MeSH Terms] OR ("hazard"[All Fields] AND "analysis"[All Fields] AND "critical"[All Fields] AND "control"[All Fields] AND "points"[All Fields]) OR "Hazard Analysis and Critical Control Points"[All Fields] OR ("safety"[All Fields] AND "analyses"[All Fields] AND "food"[All Fields])) OR ("Hazard Analysis and Critical Control Points"[MeSH Terms] OR ("hazard"[All Fields] AND "analysis"[All Fields] AND "critical"[All Fields] AND "control"[All Fields] AND "points"[All Fields]) OR "Hazard Analysis and Critical Control Points"[All Fields] OR ("safety"[All Fields] AND "analysis"[All Fields] AND "food"[All Fields])) OR "Hazard Analysis Critical Control Point"[All Fields] OR "HACCP"[All Fields] OR "HACCPs"[All Fields] OR "Hazard Analysis Critical Control Points"[All Fields] OR "Hazard Analysis And Critical Control Point"[All Fields] OR "Food Safety System"[All Fields] OR "Food Safety Systems"[All Fields] OR "safety system food"[All Fields] OR ("Hazard Analysis and Critical Control Points"[MeSH Terms] OR ("hazard"[All Fields] AND "analysis"[All Fields] AND "critical"[All Fields] AND "control"[All Fields] AND "points"[All Fields]) OR "Hazard Analysis and Critical Control Points"[All Fields] OR ("safety"[All Fields] AND "systems"[All Fields] AND "food"[All Fields])) OR "system food safety"[All Fields] OR "systems food safety"[All Fields] OR (("economics"[MeSH Terms] OR "economics"[All Fields] OR "production"[All Fields] OR "productions"[All Fields] OR "efficiency"[MeSH Terms] OR "efficiency"[All Fields] OR "productivity"[All Fields] OR "product"[All Fields] OR "product s"[All Fields] OR "productive"[All Fields] OR "productively"[All Fields] OR "productivities"[All Fields] OR "products"[All Fields]) AND ("protocol"[All Fields] OR "protocol s"[All Fields] OR "protocolized"[All Fields] OR "protocols"[All Fields])) OR (("economics"[MeSH Terms] OR "economics"[All Fields] OR "production"[All Fields] OR "productions"[All Fields] OR "efficiency"[MeSH Terms] OR "efficiency"[All Fields] OR "productivity"[All Fields] OR "product"[All Fields] OR "product s"[All Fields]	745

	OR "productive"[All Fields] OR "productively"[All Fields] OR "productivities"[All Fields] OR "products"[All Fields]) AND ("process"[All Fields] OR "processe"[All Fields] OR "processed"[All Fields] OR "processes"[All Fields] OR "processing"[All Fields] OR "processings"[All Fields])))) AND (2014:2024[pdat])	
Embase	#1 'cultured meat'/exp OR 'cultured meat'/syn<br>#2'animal free' AND 'meat'/exp OR ('animal free' AND 'meat'/syn)<br>#3 'risk'/exp OR 'risk'/syn<br>#4 'hazard assessment'/exp OR 'hazard assessment'/syn<br>#5 production AND protocol<br>#6 production AND process<br>#1 OR #2<br>#3 OR #4 OR #5 OR #6<br>#7 AND #8	133
CENTRAL (Cochrane Library)	MeSH descriptor: [In Vitro Meat] explode all trees<br>MeSH descriptor: [Animal-free meat] explode all trees<br>MeSH descriptor: [Artificial flesh] explode all trees<br>MeSH descriptor: [Risk] explode all trees<br>MeSH descriptor: [Hazard Analysis and Critical Control Points] explode all trees	0
BVS	("Carne in vitro" OR "In Vitro Meat" OR "Carne in Vitro" OR (animal-free meat) OR (artificial flesh)) AND ("Risco" OR "Risk" OR "Riesgo" OR "Risque" OR "Análise de Perigos e Pontos Críticos de Controle" OR "Hazard Analysis and Critical Control Points" OR "Análisis de Peligros y Puntos de Control Críticos" OR "Analyse des risques et maitrise des points critiques" OR (production protocol) OR (production process)) AND (collection:("06-national/BR" OR "05-specialized") OR db:("LILACS" OR "MEDLINE")) AND (db:("LILACS" OR "SES-SP"))	14
Web of science	"In Vitro Meat" OR (animal-free meat) OR (artificial flesh) (All Fields) and "risso" OR "Risk" OR "Hazard Analysis and Critical Control Points" OR (production protocol) OR (production process) (All Fields)	100
Scopus	(TITLE-ABS-KEY ("In Vitro Meat" OR (animal-free AND meat) OR (artificial AND flesh)) AND TITLE-ABS-KEY ("Risk" OR "Hazard Analysis and Critical Control Points" OR (production AND protocol) OR (production AND process))) AND PUBYEAR > 2012	93
Google scholar (limitado aos 100 primeiros	"In Vitro Meat" AND ("Risk" OR "Hazard Analysis and Critical Control Points" OR production)	100
	**TOTAL**	1185
